# Loneliness, social isolation, and living alone: a comprehensive systematic review, meta-analysis, and meta-regression of mortality risks in older adults

**DOI:** 10.1007/s40520-024-02925-1

**Published:** 2025-01-21

**Authors:** Agni Nakou, Elena Dragioti, Nikolaos-Stefanos Bastas, Nektaria Zagorianakou, Varvara Kakaidi, Dimitrios Tsartsalis, Stefanos Mantzoukas, Fotios Tatsis, Nicola Veronese, Marco Solmi, Mary Gouva

**Affiliations:** 1 Research Laboratory Psychology of Patients, Families, and Health Professionals, Department of Nursing, School of Health Sciences, University of Ioannina, Ioannina, Greece; 2 Research Laboratory Integrated Care, Health & Well-being, Department of Nursing, School of Health Sciences, University of Ioannina, Ioannina, Greece; 3 Department of Clinical Physiology, Sundsvall Hospital, Sundsvall, Sweden; 4 Faculty of Medicine, School of Health Sciences, University of Ioannina, Ioannina, Greece; 5 Unit of Geriatrics, Department of Internal Medicine and Geriatrics, University of Palermo, Palermo, Italy; 6 SCIENCES lab, Department of Psychiatry, University of Ottawa, Ottawa, ON Canada; 7 Regional Centre for the Treatment of Eating Disorders and On Track: The Champlain First Episode Psychosis Program, Department of Mental Health, The Ottawa Hospital, Ottawa, ON Canada; 8 Ottawa Hospital Research Institute (OHRI) Clinical Epidemiology Program, University of Ottawa, Ottawa, ON Canada; 9 Department of Child and Adolescent Psychiatry, Charité Universitätsmedizin, Berlin, Germany; 10 Mental Health Department, The Ottawa Hospital, 501 Smyth Road, Ottawa, ON Canada

**Keywords:** Loneliness, Social isolation, Mortality, Older adults, Systematic review, Meta-analysis

## Abstract

Loneliness, social isolation, and living alone are significant risk factors for mortality, particularly in older adults. This systematic review and meta-analysis aimed to quantify their associations with all-cause and cause-specific mortality in older adults, broadening previous research by including more social factors. Comprehensive searches were conducted in PubMed, APA PsycINFO, and CINAHL until December 31, 2023, following PRISMA 2020 and MOOSE guidelines. Studies included were prospective cohort or longitudinal studies examining the relationship between loneliness, social isolation, living alone, and mortality. Quality was assessed using the Newcastle-Ottawa Scale. Meta-analyses used random-effects models with the Restricted Maximum Likelihood method. Subgroup and meta-regression analyses explored the relationships further. Of 11,964 identified studies, 86 met the inclusion criteria. Loneliness was associated with increased all-cause mortality (HR 1.14, 95% CI 1.10–1.18), with substantial heterogeneity (I² = 84.0%). Similar associations were found for social isolation (HR 1.35, 95% CI 1.27–1.43) and living alone (HR 1.21, 95% CI 1.13–1.30). Subgroup analyses revealed variations based on factors like sex, age, region, chronic diseases, and study quality. Meta-regression identified longer follow-up, female sex, validated social network indices, adjustments for cognitive function, and study quality as significant predictors of mortality risks. These findings highlight the need for public health interventions to address these social factors and improve health outcomes in older adults. However, further research is needed due to variability and heterogeneity across studies. Also studying the cumulative effect of these factors on mortality risks will be of considerable interest.

**Supplementary Information:**

The online version contains supplementary material available at 10.1007/s40520-024-02925-1.

## Introduction

As global demographics trend toward aging populations [1], the pervasive issue of social dynamics like loneliness and social isolation in older adults has garnered increasing attention due to their potential impact on health outcomes [1–4]. Loneliness refers to the subjective, distressing experience of feeling socially isolated or disconnected, even when one is not physically alone [1]. This experience is inherently subjective, shaped by an individual’s expectations and perceived inadequacy in their social relationships [5]. In contrast, social isolation is an objective state defined by a lack of meaningful social connections or participation in social activities [1]. Finally, living alone represents a straightforward demographic measure of solitary living arrangements [1, 6], which often intersects with social isolation and loneliness but remains a distinct construct [7, 8]. These three dimensions, while interrelated [9–12], represent unique aspects of social well-being [4], each with potentially differing implications for health outcomes [13, 14].

Despite their distinct definitions, these constructs often overlap in the lives of older adults, particularly among those who live alone. Living alone, increasingly common among aging populations due to socio-demographic shifts like declining marriage rates, rising divorce [1, 6], and longer life expectancy [9–12], serves as a tangible marker of potential social isolation [7, 8]. However, it is critical to underscore that living alone does not inherently equate to loneliness or social isolation [4]. For instance, an older adult who lives alone may maintain an active social life, while another living with others may still feel profoundly lonely. Conversely, social isolation does not always result in loneliness, as some individuals may not perceive their limited social connections as problematic. These nuanced relationships highlight the need to differentiate between these concepts and their measurement approaches to fully understand their respective impacts on health outcomes in older adults. Such distinctions are crucial not only conceptually but also methodologically. Loneliness is predominantly assessed through self-reported measures, such as the UCLA Loneliness Scale, which capture subjective experiences and perceptions of social disconnection. Social isolation, while sometimes assessed subjectively, is often measured using objective indicators such as social network size or the frequency of social interactions. In contrast, living alone is typically captured through objective demographic data, such as census records or self-reports of household composition, providing a structural perspective on solitary living arrangements.

The ramifications of these social constructs extend far beyond emotional well-being, influencing both physical and mental health outcomes [3, 15, 16]. Evidence showed that loneliness, social isolation, and living alone have been associated prospectively with decline in gait speed [13], with increased mortality [7, 10, 14, 17–19], incidence coronary heart disease [20–22], conditions that reduce immune system response [3, 23, 24] and functional decline [13, 25, 26] in older people. Notably, elevated levels of loneliness, but not necessarily social isolation, have been linked to an increased likelihood of physical frailty [27, 28]. Moreover, living alone and loneliness are both independently associated with higher risk of mortality [29]. Social isolation, meanwhile, has been identified as a predictor of mortality for both genders, comparably influential as smoking and high blood pressure [19]. Living alone is particularly associated with increased mortality in individuals below the age of 65, but this association does not hold for those above 75 years of age [6]. Furthermore, these social dynamics is associated with anxiety, psychosis, substance abuse [3, 23], alcoholism [30], depression [31–33], cognitive impairment [23, 26, 34], decreased subjective well-being [3], sleep disorders [23], reduced quality of life [35] and suicidal tendencies [36].

However, while some meta-analyses have underscored the adverse effects of social dynamics on health outcomes in adults [6, 7, 10, 17, 18, 22, 37–39], the precise relationship between loneliness, social isolation, and living alone and mortality remains an area of ongoing inquiry and discussion within scientific circles [3]. Firstly, no studies to date have simultaneously investigated all three factors—loneliness, social isolation, and living alone—in the context of older adults. Existing research has predominantly examined these constructs separately [6, 18, 38, 39] or in a restricted combination [7, 17, 37]. Only one recent meta-analysis has examined the association between these factors and mortality risk, yet its focus was narrowed to individuals with pre-existing cardiovascular conditions [10]. Secondly, a significant number of these reviews have not been specifically dedicated to older adults [6, 7, 10, 22, 37–39], resulting in a noticeable underrepresentation of older adults in their findings, particularly with regard to sex-based differences [6, 18]. The increasing prevalence of these phenomena in modern society [40], coupled with their potential health implications, highlights the need for an updated, focused analysis to clarify the individual contributions of loneliness, social isolation, and living alone to mortality risk in this vulnerable population, as further emphasized by the work of Schutter et al. [17].

Given this background, we aimed to examine and quantify the relationship between these social dynamics and mortality risks (all-cause and disease-specific) in older adults. While this study did not explore their combined or interactive effects, it lays the groundwork for future research to investigate how these factors may work together to influence health outcomes. The urgency for a thorough meta-analysis becomes clear in light of the diverse characteristics of studies previously included in such analyses– ranging from geographical origins of data, methods of measurement, duration of follow-up, to critical covariates like health status and mental health levels [10, 18]. Such an approach is pivotal to attain a more intricate and detailed understanding of these complex associations in older adults.

## Methods

This systematic review with meta-analysis was undertaken in accordance with the guidelines set forth by the Preferred Reporting Items for Systematic Reviews and Meta-Analyses (PRISMA) of 2020 [41], as well as the protocols outlined for the Reporting of Meta-Analyses of Observational Studies (MOOSE) [42]. The predetermined protocol was deposited on the Open Science Framework and is readily accessible at the following link: https://osf.io/3qbd8.

### Data sources and searches

This study entailed a comprehensive inquiry conducted across three electronic databases (PubMed, APA PsycINFO and CINAHL [via EBSCOhost]) to identify articles examining the relationship between loneliness, social isolation, living alone, and mortality from the inception of these databases until December 31, 2023. We employed a combination of keywords, synonyms, and MeSH terms associated with concepts including loneliness, social isolation, living alone, mortality, and older adults. We utilized Boolean operators ‘AND’ and ‘OR’ in our search strategy. The full search strategies are detailed in the Supplementary Material (see the ‘Search Strategy’ section). We did not impose any limitations regarding language, country of origin, year of publication, or specific characteristics such as residential setting, physical condition, or age of older adults. However, we did apply limitations related to study design and human subject restrictions. We also reviewed the references of the retrieved articles to identify any relevant studies that may have been missed in the electronic database search.

### Study selection

Employing the Population (P: older adults), Exposure (E: loneliness, social isolation, and living alone), Outcome (O: all-cause or cause-specific mortality) paradigm, our research carefully curated study selections. We exclusively included studies that: (a) constituted prospective cohort or longitudinal investigations; (b) explored the association between loneliness, social isolation, and living alone and mortality (be it all-cause or cause-specific); (c) included participants aged 65 and above, dwelling in home environments, residential care establishments, or hospitals; and (d) were published in peer-reviewed journals. Inclusion criteria also extended to studies involving individuals aged between 50 and 65 years, provided that at least 75% of participants were over 65. Research exploring dimensions beyond loneliness or perceived social isolation, such as network size, living arrangement etc., were also included (as delineated below in differences between protocol and review). The primary outcome of interest was all-cause mortality, while disease-specific mortality (e.g., cancer mortality) was considered as secondary outcome. Studies that evaluated the association between loneliness, social isolation, living alone and deaths by suicide, injury, or accidents were similarly considered.

Exclusions were reserved for studies deviating from prospective cohort or longitudinal design and for those where most participants were under the mean age of 65 years. Articles failing to explicitly probe the association between loneliness, social isolation, living alone, and mortality were excluded. These were, for example, studies that examined variables such as social support as opposed to loneliness and social isolation [10]. Likewise, research that did not position loneliness, social isolation, and living alone as independent variables, or mortality as a dependent variable, were omitted. This encompassed, for instance, validations of questionnaires or studies exploring correlations through moderation analysis. Studies that did not report association effect metrics, such as hazard ratios (HRs) or relative risk (RRs) or similar time-dependent estimates or provide sufficient data to calculate such metrics were also excluded. Moreover, despite not imposing language restrictions on our search, studies in languages other than English were, regrettably, sidelined due to constraints in accurate translation resources.

### Screening

The retrieved search results were transferred to the Mendeley (1.9.8) citation management software, where duplicate entries were removed. These refined results were subsequently ported into an Excel spreadsheet, primed for enhanced scrutiny. The first phase of evaluation, conducted by two independent reviewers (AN and ED), involved a thorough inspection of titles and abstracts. The retrieved full texts were then examined independently by the same pair of reviewers using our predefined inclusion criteria. In case of disagreement, a third impartial reviewer (MG) was consulted.

### Data extraction

Three independent reviewers (AN, ED, and NB) extracted relevant information from the included studies into a pre-designed Excel sheet. We recorded various data such as the Digital Object Identifier (DOI) and PubMed Identifier (PMID) of the study, first author, year of publication, country, study design, sample size, mean age or age range, and settings (e.g., at home, etc., if possible). Additionally, we recorded any reported underlying physical or mental disorder, variables that were controlled for, exposures (such as loneliness, social isolation, and living alone), and the assessment tools used. Furthermore, we captured information on the type of mortality (all-cause or cause specific mortality) and association effect metrics (e.g., HR, etc.), along with the 95% confidence intervals (95% CI). In cases where the 95% CI was not reported, the p-value was extracted to estimate it. Estimates were extracted, with a primary focus on maximally adjusted estimates whenever available. The final data underwent independent checking and assessment by another author (MG).

### Quality assessment

The Newcastle-Ottawa Scale (NOS) was used to assess the risk of bias and the quality of the included studies. It is particularly applicable for evaluating the quality of cohort and case-control studies [43]. The NOS employs a star system, with a maximum of 9 stars, to evaluate studies across three domains: selection, comparability, and outcome. Generally, a higher score (more stars) indicates better quality and a lower risk of bias [43].

### Statistical analysis

Data were analysed by exposure type (loneliness, social isolation, living alone) and outcome type (all cause and cause specific mortality). We pooled OR, RR, HR as summary HRs, acknowledging that for some studies with lower absolute mortality rates, these measures approximate the same association. While mortality is not rare in all included studies, the methodological consistency across designs and the similarity of relative effect estimates in most cases justify this approach. HR values above 1 indicate an increased risk of mortality, while HR values below 1 indicate a decreased risk of mortality. In cases where a single study reported multiple results for different subgroups (e.g., stratified by sex or age), these results were not pooled into a single effect size. Instead, subgroup analyses were conducted wherever feasible to account for the variability within the study while maintaining the integrity of the reported outcomes. To estimate the summary HRs, random-effects models were employed using the Restricted Maximum Likelihood (REML) method, because heterogeneity across studies was expected [44]. The REML method is an advanced technique used for estimating the variance components in such models, especially when dealing with different study sizes and numbers [45]. Heterogeneity between studies was assessed using I² and tau² statistics, along with 95% prediction intervals (PIs), to estimate the range where the effect sizes of future studies are likely to fall [46]. Unlike 95%CIs, which estimate where the true effect size lies with a certain degree of confidence, PIs account for the variability between studies and offer a more practical insight into the real-world implications of the results [47]. For analyses involving 10 or more studies, funnel plots and the Egger test were employed to evaluate the presence of publication bias [48, 49]. Cumulative and leave-one-out sensitivity meta-analyses were also performed to assess the robustness of the results where possible [50].

We conducted subgroup analyses based on various dimensions and variables to approach the problem of heterogeneity. These included the dimension of loneliness (i.e., this only applied to the exposure of loneliness; emotional loneliness and social loneliness), sex (both sexes, male, and female), age groups (≥ 50–64, ≥ 65, ≥75 years), and population (general, those with cardiovascular disease (CVD), Type 2 Diabetes Mellitus (T2DM), etc.,). We also differentiated based on the instrument used for measuring loneliness, social isolation, and living alone (validated scales and other single-item scales), geographical location (Europe, North America, and other regions), follow-up duration (< 5 years, ≥ 5 years), and NOS score (≥ 7 as high quality, ≤ 6 as lower quality). Moreover, our analysis considered whether studies adjusted for potential confounders, including income, mental symptoms, physical symptoms, and cognitive function. Adjusting physical symptoms is particularly relevant as they may act as confounders or mediators in the relationship between social constructs and mortality. For example, physical symptoms may both exacerbate social isolation and directly increase mortality risk, making it critical to disentangle their effects to better understand the independent contribution of loneliness, social isolation, and living alone to mortality outcomes.

Finally, random-effects meta-regression analyses were conducted to explore the potential moderating effects of the mean age of the participants, sex (female), geographical region, NOS assessment scores, follow-up duration, use of validated instruments, population type, and adjusted estimates of the observed associations for the primary outcome [51]. All statistical analyses were performed using STATA 17.0 (StataCorp, College Station, Texas 77845 USA) software.

### Differences between protocol and review

In our review, expanded the scope to include social isolation and living alone alongside loneliness to capture a more comprehensive understanding of how different, yet interrelated, aspects of social experience affect mortality [10]. We also expanded our database search to December 2023 for the latest evidence. The review and protocol included both subgroup and meta-regression analyses to examine potential moderating effects. However, the analysis details of the review varied slightly based on the data reported, with a focus on the primary outcome. Additionally, no studies were found that examined deaths by suicide, injury, or accidents.

## Results

### Search results

**Fig. 1:**
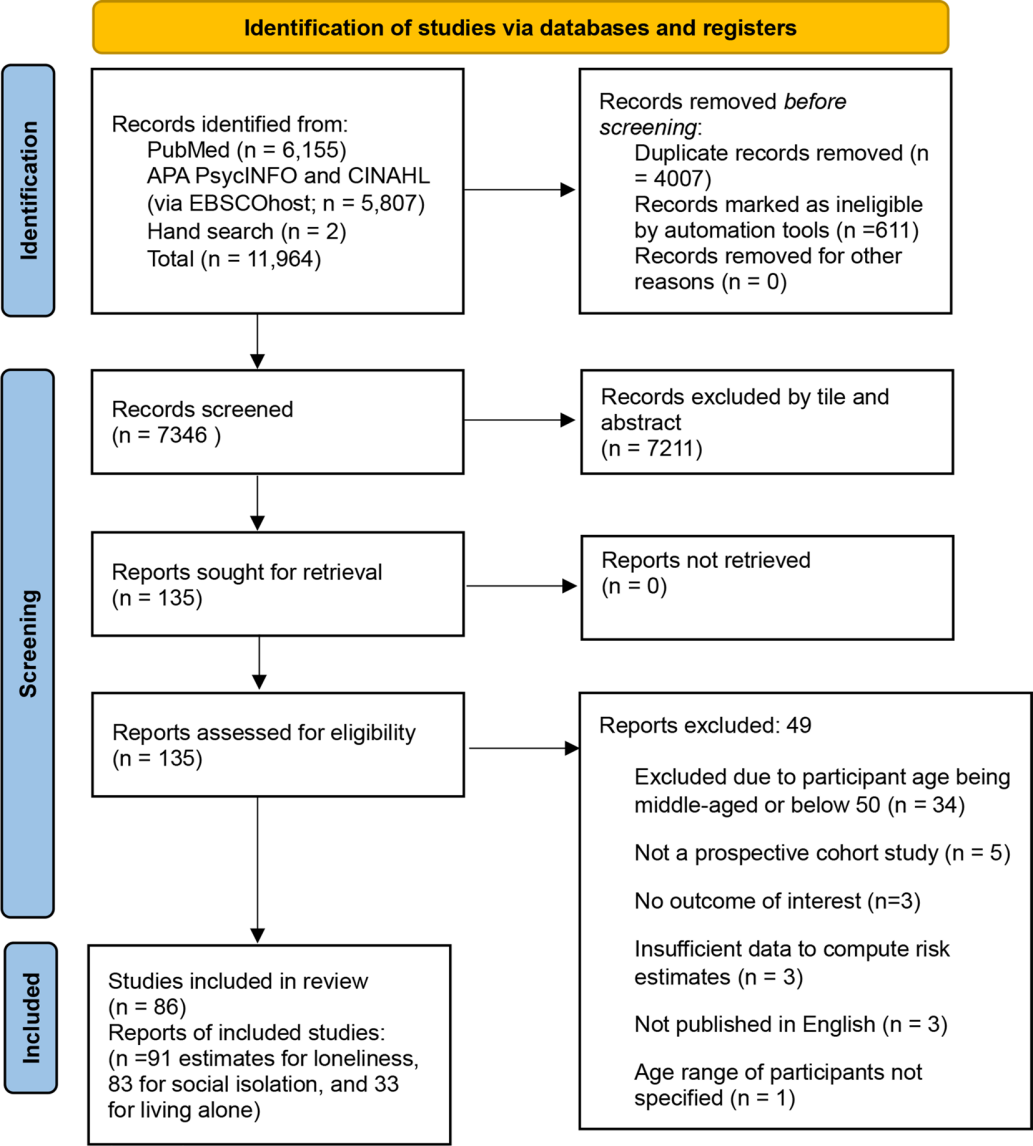
Flow diagram for the study selection process

Figure 1 delineates the study selection process. Initially, a search yielded 11,964 studies. Upon the removal of 4,618 duplicates, 7,346 studies underwent screening. Of these, 135 full texts were thoroughly assessed, leading to the exclusion of 49; reasons for this exclusion are detailed in Fig. 1, with an exhaustive list of excluded studies and their respective reasons provided in the supplementary material (Table S1). A total of 86 prospective cohort or longitudinal studies were finally included [14, 25, 26, 29, 52–133].

### Characteristics of included studies

Study characteristics are summarized in Tables S2, S3, and S4 in the supplementary material. The eligible studies were published between 1989 [78] and 2023 [71, 76, 89, 100, 132]. The majority of the studies (*n* = 73; 84.9%) targeted the general older population. Among them, 13 studies specifically concentrated on individuals diagnosed with CVD [55, 58, 73, 84, 93, 95, 108, 130], while others focused on populations with cancer [63, 85, 86], T2DM [89] and hip fracture [62]. The participants’ ages ranged from 50 to 103 years [92] and many studies had separate entries for different groups (e.g., for female and male groups or for cohorts or measurements, or for age groups) [53, 55, 56, 58, 64–66, 69, 70, 75, 78, 81, 83, 85, 86, 88, 90, 97–100, 107, 109, 110, 114, 122, 127, 131, 132]. Most studies (*n* = 82; 95.3%) controlled for potential confounding factors, with the exception of four studies [92, 99, 103, 113]. Notably, there was considerable diversity in the scope and nature of the variables accounted for as confounders across these studies. Fifty-four studies provided data on loneliness, 37 on social isolation, and 17 on living alone.

### Quality assessment

A total of 69 studies, constituting 80.2% of the sample, were classified as high quality, while 17 studies, constituting 19.8% of the total, were classified as moderate (Table S5 in the supplementary material).

### Meta-analysis of the association between loneliness and mortality in older adults

The association between loneliness and mortality was examined in 54 individual studies, which provided 91 estimates and included a total of 310,095 participants. The studies were categorized into three groups based on the outcomes of interest: all-cause mortality [14, 25, 26, 29, 54–61, 63–66, 68, 70–72, 77, 78, 87–89, 91, 92, 94, 96, 97, 99–101, 103, 104, 106, 113, 115, 116, 118–121, 123–126, 129–133] (52 studies, 82 estimates, *n* = 308, 948), cardiovascular disease (CVD) mortality [14, 77, 89, 99, 102, 121] (6 studies, 8 estimates, *n* = 37104), and cancer mortality [14] (1 study, 1 estimate, *n* = 6915).

#### Primary outcome

##### All-cause mortality

**Fig. 2:**
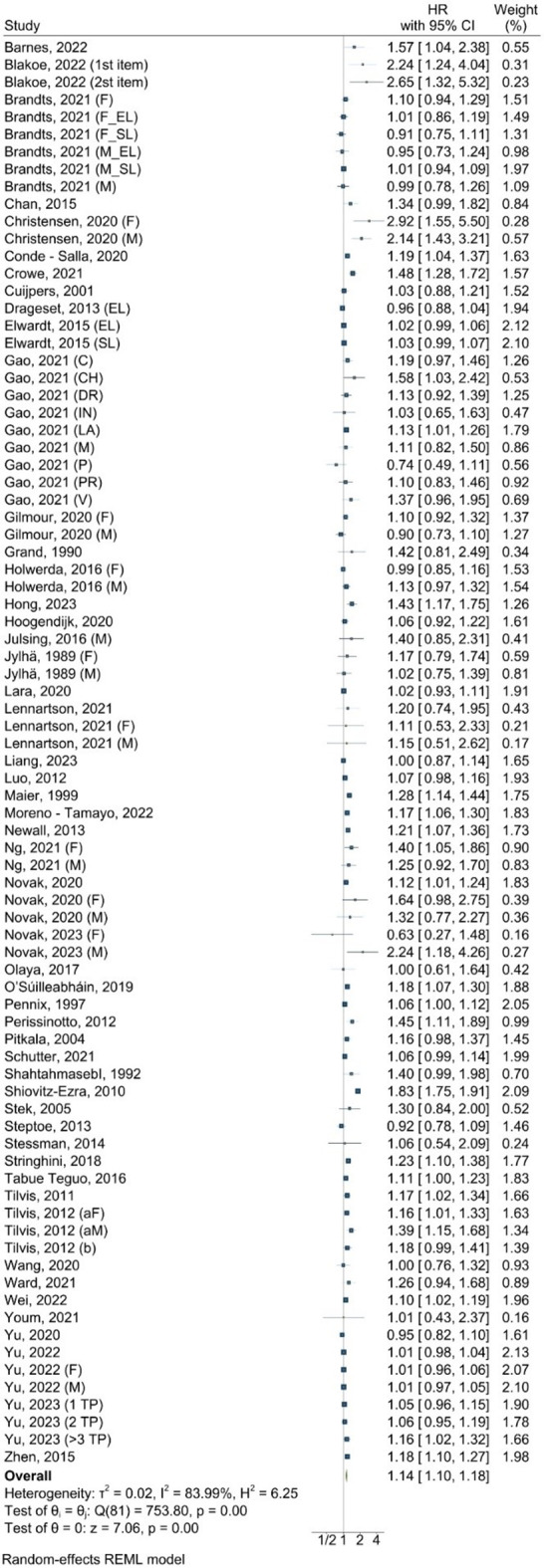
Forest plot for the association between loneliness and all-cause mortality Notes: F = female, M = male. The x-axis represents hazard ratios (HRs), where values above 1 indicate an increased likelihood of mortality, and values below 1 indicate a decreased likelihood of mortality

For the association between loneliness and all-cause mortality, the combined HR was 1.14 (95% CI: 1.10–1.18, *P* = 0.00), indicating a significant increase in all-cause mortality risk (Fig. 2). The individual study HRs ranged from 0.62 [100] to 2.92 [58], both in females, suggesting variability in effect sizes. The heterogeneity was substantial (I^2^ = 84.0%, tau^2^ = 0.02, *P* < 0.001). The 95% PI ranged from 0.89 to 1.46, reflecting the variation in the observed effects and the Egger test implied a high likelihood of publication bias (*P* = 0.00, supplementary figure S1).

The cumulative meta-analysis showed that the HRs across studies were fairly consistent, generally ranging around 1.10 to 1.20 (supplementary figure S2), with only early studies (e.g., in 1989–1997) showing non-significant results (*P* > 0.05). Similarly, the leave-one-out sensitivity meta-analysis also produced HRs that ranged narrowly (*P* < 0.001), reflecting the robustness of the meta-analysis results (supplementary figure S3).

#### Secondary outcomes

##### CVD mortality

For the association between loneliness and CVD mortality (supplementary figure S4), the combined HR was 1.30 (95% CI: 1.09–1.54, *P* = 0.00). The studies exhibited moderate heterogeneity (I^2^ = 44.4%, tau2 = 0.02, *P* = 0.08). Notably, the study conducted by Novak et al. [99] reported the highest HR at 2.25 in females, whereas the lowest HR of 1.03 was observed in the study by Liang et al. [89]. The 95% PI was between 0.85 and 1.97, suggesting variability in the effect sizes that could be expected in future studies. Due to the insufficient number of studies (i.e., fewer than ten), a formal assessment for publication bias was deemed not applicable. The cumulative meta-analysis indicated a slight increase in HRs over time, with more recent studies demonstrating statistically significant results (supplementary figure S5). Additionally, leave-one-out sensitivity analysis revealed that the exclusion of any single study did not substantially alter the overall effect size or its statistical significance (supplementary figure S6).

##### Cancer mortality

Only one study [14] was included for the association between loneliness and cancer mortality (Supplementary figure S4), reporting an HR of 1.02 (95% CI: 0.68–1.53, *P* = 0.92). Due to insufficient number of studies, heterogeneity measures, prediction intervals, publication bias, cumulative and leave-one-out meta-analyses were not applicable.

### Meta-analysis of the association between social isolation and mortality in older adults

The association between social isolation and mortality was examined in 37 individual studies, which provided 83 estimates and included a total of 357,457 participants. The studies were categorized into four groups based on the outcomes of interest: all-cause mortality [14, 54, 57, 63, 64, 66, 69, 71–73, 75, 78, 79, 82–86, 88–90, 93–95, 108–111, 114, 117, 125, 128–131] (37 studies, 61 estimates, *n* = 233,279), CVD mortality [14, 76, 127, 128] (4 studies, 8 estimates, *n* = 63,434), cancer mortality [14, 85, 86, 127, 128] (5 studies, 10 estimates, *n* = 54,759) and other mortality [127] (1 study, 4 estimates, *n* = 30,430).

#### Primary outcome

##### All-cause mortality

**Fig. 3:**
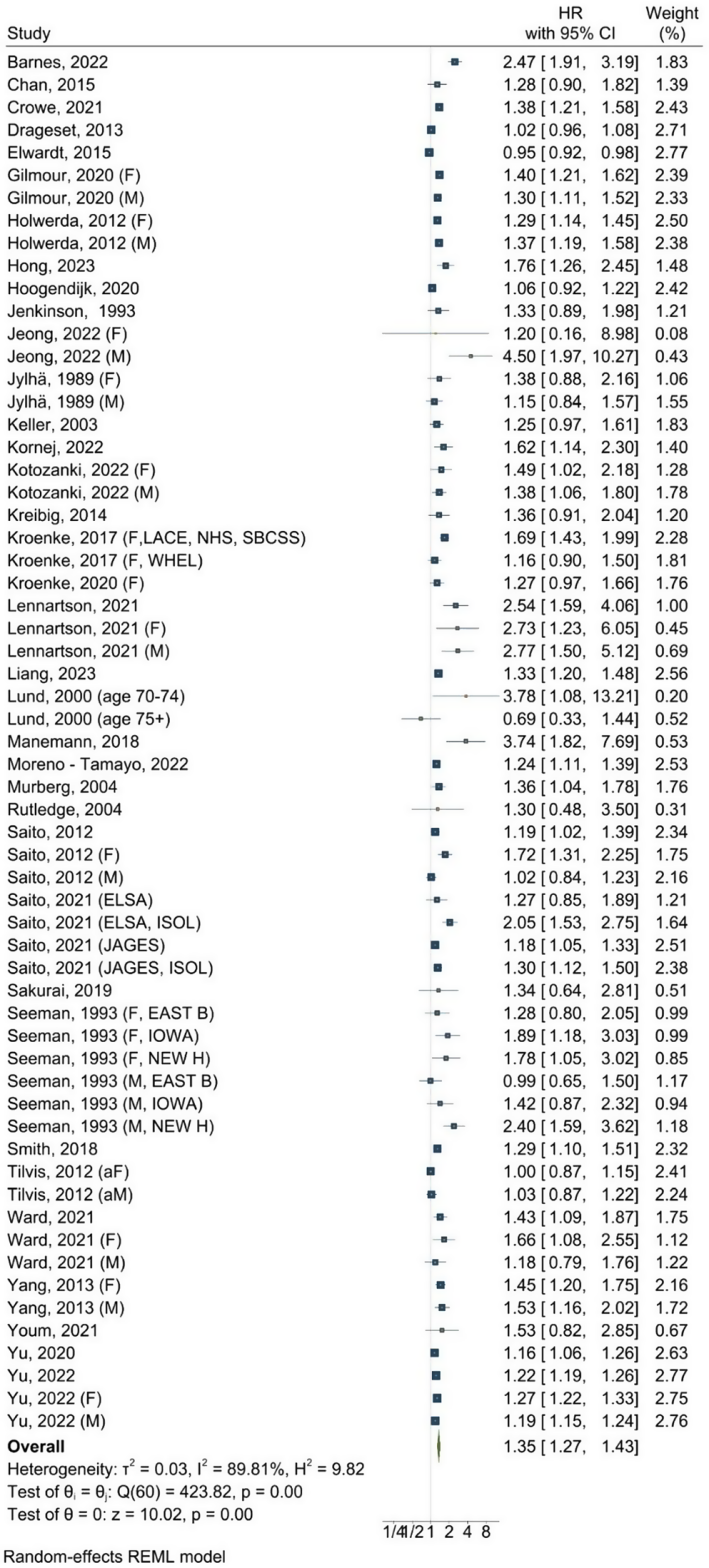
Forest plot for the association between social isolation and all-cause mortality Notes: F = female, M = male. The x-axis represents hazard ratios (HRs), where values above 1 indicate an increased likelihood of mortality, and values below 1 indicate a decreased likelihood of mortality

For the association between social isolation and all-cause mortality, the combined HR was 1.35 (95% CI: 1.27–1.43, *P* = 0.00), indicating a significant increase in all-cause mortality risk (Fig. 3). The individual study HRs varied notably across studies, with Lennartson et al. [88] reporting a high of 2.77 in females and Lund et al. [90] reporting a low of 0.69 in individuals aged 75 and older. The heterogeneity was substantial (I^2^ = 89.8%, tau2 = 0.03, *P* < 0.001).

The 95% PI ranged from 0.94 to 1.94, reflecting the variation in the observed effects. The presence of publication bias was suggested by the results of the Egger test (*P* = 0.00, supplementary figure S7). The HRs in more recent studies (2020s) appeared to be slightly higher than earlier studies in the cumulative meta-analysis (supplementary figure S8), whereas the leave-one-out meta-sensitivity analysis showed that HRs remained consistently above 1.30 (*P* < 0.001) across all scenarios (supplementary figure S9).

#### Secondary outcomes

##### CVD mortality

For the association between social isolation and CVD mortality, the combined HR was 1.37 (95% CI: 1.19–1.56, *P* = 0.00, as shown in supplementary figure S10), indicating a significant association. Moderate heterogeneity was observed (I^2^ = 65.2%, tau2 = 0.02, *P* = 0.001). The HRs in study by Wang et al. [127] ranged from 1.05 to 1.61. The 95% PI was between 0.91 and 2.03, indicating some variability in potential future studies. Due to the insufficient number of studies (i.e., fewer than ten), a formal assessment for publication bias was deemed not applicable. The HRs tend to be increased slightly over the years (supplementary figure S11) and the consistency in HRs across different leave-one-out sensitivity scenarios further strengthens the evidence for a stable association (supplementary figure S12).

##### Cancer mortality

For the association between social isolation and cancer mortality (supplementary figure S10), the combined HR was 1.13 (95% CI: 0.98–1.29, *P* = 0.09) with moderate heterogeneity (I^2^ = 67.2%, tau2 = 0.03, *P* < 0.001). The 95% PI ranged from 0.75 to 1.71, suggesting some variability in the effect sizes in future studies. Due to the insufficient number of studies (i.e., fewer than ten), a formal assessment for publication bias was deemed not applicable. The trend over the years showed no clear increase or decrease in HRs, suggesting a stable but not strongly pronounced effect over time (supplementary figure S13). Leave-one-out sensitivity analysis revealed that the overall HRs fluctuated slightly but generally remained within a similar range (supplementary figure S14).

##### Other mortality types

For the association between social isolation and other mortality types (supplementary figure S10), the combined HR was 1.24 (95% CI: 1.03–1.49, *P* = 0.03), with moderate heterogeneity observed (I^2^ = 69.2%, tau2 = 0.02, *P* = 0.01). The 95% PI ranged from 0.57 to 2.67, indicating substantial variability in future studies. Due to insufficient number of studies, publication bias, cumulative and leave-one-out meta-analyses were not applicable.

### Meta-analysis of the Association between Living Alone and mortality in older adults

A total of 17 individual studies with 33 estimates and a total of 93,011 participants were included in this meta-analysis to assess the impact of living alone on mortality. The analysis included 17 studies on all-cause mortality [26, 52, 53, 57, 62, 67, 74, 76, 78, 80, 81, 98, 100, 105, 107, 112, 122] (29 estimates, *n* = 86,553), two studies focused on CVD mortality [74, 80] (2 estimates, *n* = 4868), one on cancer mortality [80] (1 estimate, *n* = 1522), and one on non-CVD, non-cancer mortality [80] (1 estimate, *n* = 1522).

#### Primary outcome

##### All-cause mortality

**Fig. 4:**
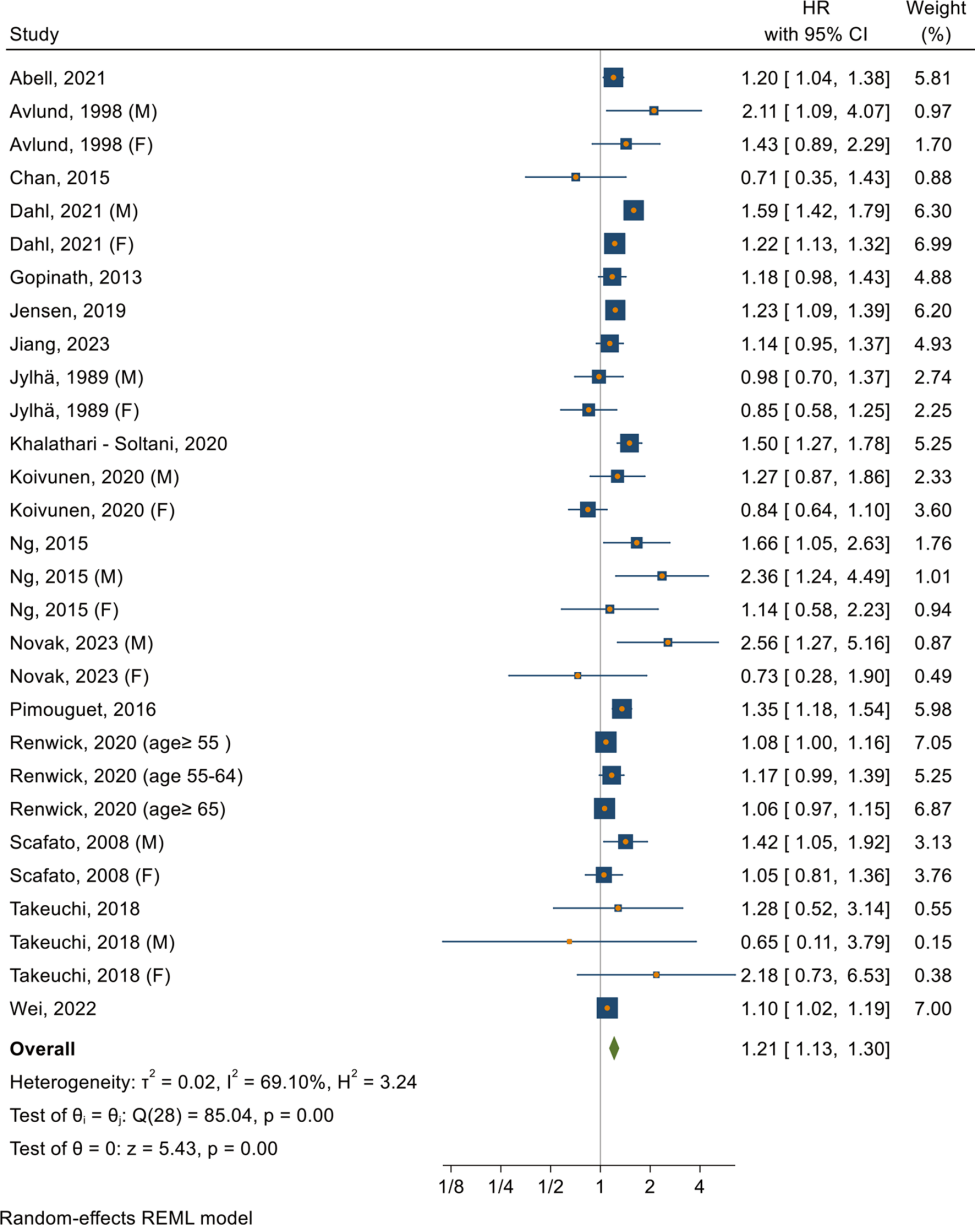
Forest plot for the association between living alone and all-cause mortality Notes: F = female, M = male. The x-axis represents hazard ratios (HRs), where values above 1 indicate an increased likelihood of mortality, and values below 1 indicate a decreased likelihood of mortality

For the association between living alone and all-cause mortality (Fig. 4), the combined HR was 1.21 (95% CI: 1.13–1.30, *P* < 0.001), indicating a significantly increased all-cause mortality risk. The studies presented a wide range of HRs. Notably, Novak et al. [100] reported the highest HR of 2.56 in males, while Takeuchi et al. [122] also reported the lowest HR of 0.65 in males. The analysis revealed moderate heterogeneity among the studies (I^2^ = 69.1%, tau2 = 0.02, *P* < 0.001). The 95% PI for the combined HR was between 0.92 and 1.59, suggesting that future studies are likely to report effect sizes within this range. No evidence of publication bias was found (*P* = 0.43, supplementary figure S15). The HRs in the most recent studies (2021–2023) were quite stable while the earlier studies (1989–2008) were generally closer to 1.00 and were not significant (supplementary figure S16). The leave-one-out sensitivity analysis showed that the omission of any single study did not lead to a significant change in the overall HR (supplementary figure S17).

#### Secondary outcomes

##### CVD mortality

Two studies [74, 80] were included for the association between living alone and CVD mortality (supplementary figure S18), reporting an HR of 1.37 (95% CI: 1.17–1.61, *P* < 0.001), with no evidence of heterogeneity among the studies (I^2^ = 0.00%, tau2 = 0.00, *P* = 0.86). Due to insufficient number of studies prediction intervals, publication bias, cumulative and leave-one-out meta-analyses were not applicable.

##### Cancer mortality

Only one study [80] was included for the association between living alone and cancer mortality (supplementary figure S18), reporting an HR of 1.36 (95% CI: 0.98–1.89, *P* = 0.07). Due to insufficient number of studies, heterogeneity measures, prediction intervals, publication bias, cumulative and leave-one-out meta-analyses were not applicable.

##### Other mortality types

Only one study [80] was also included for the association between living alone and non-CVD, non-cancer mortality (supplementary figure S18), reporting an HR of 1.76 (95% CI: 1.29–2.40, *P* < 0.001). Due to insufficient number of studies, heterogeneity measures, prediction intervals, publication bias, cumulative and leave-one-out meta-analyses were not applicable.

### Subgroup analysis

Tables 1 and 2, and 3 present the results of various subgroup analyses. In the analysis of loneliness and all-cause mortality (Table 1), individuals with CVD showed the highest HR at 1.91 (95% CI: 1.22–3.01). Studies using validated loneliness scales revealed a lower HR of 1.10 (95% CI: 1.06–1.15) in contrast to those with single-item measures. A higher HR of 1.22 (95% CI: 1.13–1.32) was associated with follow-up periods of less than 5 years compared to longer follow-ups. Studies that accounted for cognitive function reported a lower HR of 1.08 (95% CI: 1.04–1.12) than those not adjusted.

**Table 1:** Summary HRs in various subgroup analyses for the association between loneliness and all-cause mortality

Category	Subgroup	No. of Studies/ estimates	Sample Size	HR (95% CI) random effects	I² (%)	*P*	Group Differences (Cochran’s Q test *P*)
Dimension of loneliness	Emotional loneliness	4	5248	1.00 (0.96–1.05)	13.9	0.85	0.52
	Social loneliness	3	5021	1.02 (0.99–1.06)	0.0	0.21	
Sex	Both	55	266,048	1.15 (1.10–1.20)	86.0	0.00	0.22
	Male	14	42,562	1.14 (1.02–1.28)	80.0	0.02	
	Female	13	36,660	1.07 (1.00-1.14)	31.8	0.04	
Age	Age ≥ 50–65	23	141,393	1.15 (1.07–1.24)	92.5	0.00	0.43
	Age ≥ 65	59	135,822	1.12 (1.08–1.26)	64.0	0.00	
	Age ≥ 75	11	47,188	1.10 (1.02–1.18)	81.9	0.01	
Population	General	75	266,877	1.13 (1.09–1.17)	82.9	0.00	0.00
	CVD	5	21,879	1.91 (1.22–3.01)	81.8	0.01	
	T2DM	1	19,360	1.00 (0.87–1.15)	NA	0.88	
	Cancer: Present vs. Absent	1	227	0.96 (0.89–1.05)	NA	0.33	
Instrument used	Validated Loneliness Scales	38	169,820	1.10 (1.06–1.15)	74.9	0.00	0.08
	Other (single items)	44	138,523	1.18 (1.11–1.25)	86.9	0.00	
Country	Europe	48	130,138	1.10 (1.07–1.14)	58.9	0.00	0.13
	North America	12	57,892	1.24 (1.10–1.40)	91.8	0.00	
	Other*	22	112,331	1.09 (1.04–1.14)	63.9	0.00	
Follow-up	< 5 years	31	188,735	1.22 (1.13–1.32)	92.8	0.00	0.01
	≥ 5 years	51	119,608	1.10 (1.06–1.13)	63.0	0.00	
NOS scale score	≥ 7	61	249,209	1.12 (1.07–1.16)	86.2	0.00	0.06
	≤ 6	21	59,134	1.21 (1.13–1.30)	62.0	0.00	
Adjusted for income	Yes	18	110,430	1.14 (1.10–1.18)	60.5	0.00	0.91
	No	64	197,913	1.13 (1.09–1.18)	86.4	0.00	
Adjusted for mental symptoms	Yes	46	220,869	1.13 (1.07–1.19)	89.4	0.00	0.61
	No	36	87,474	1.15 (1.10–1.19)	59.8	0.00	
Adjusted for physical symptoms	Yes	44	235,866	1.16 (1.09–1.23)	92.1	0.00	0.41
	No	38	72,477	1.12 (1.09–1.16)	48.8	0.00	
Adjusted for cognitive function	Yes	27	117,857	1.08 (1.04–1.12)	42.5	0.00	0.02
	No	55	190,486	1.16 (1.11–1.22)	87.4	0.00	

* The ‘Other’ region category includes countries outside Europe and North America, such as China, Israel, Korea, Mexico, and Singapore. Notes: CVD = cardiovascular disease, HR = Hazzard ratio, I^2^ = heterogeneity, NA = not applicable, NOS = Newcastle Ottawa Scale, P = p-value, T2DM = type 2 diabetes mellitus. Group differences were calculated using Cochran’s Q test for subgroup differences

When examining social isolation and all-cause mortality (Table 2), the study found a higher HR in individuals with cancer of 1.38 (95% CI: 1.09–1.75) compared to other populations. Additionally, studies with a lower NOS score (≤ 6) showed a notably high HR of 1.81 (95% CI: 1.34–2.46) in contrast to those with higher NOS scores. Geographically, North America exhibited a significant HR of 1.51 (95% CI: 1.37–1.67) than other regions.

**Table 2:** Summary HRs in various subgroup analyses for the association between social isolation and all-cause mortality

Category	Subgroup	No. of Studies/ estimates	Sample Size	HR (95% CI) random effects	I² (%)	*P*	Group Differences (Cochran’s Q test *P*)
Sex	Both	30	160,429	1.34 (1.23–1.47)	92.9	0.00	0.87
	Male	14	44,031	1.34 (1.27–1.43)	82.2	0.00	
	Female	17	65,234	1.39 (1.26–1.52)	68.6	0.00	
Age	Age ≥ 50–65	23	101,303	1.34 (1.24–1.46)	68.7	0.00	0.32
	Age ≥ 65	37	130,600	1.37 (1.25–1.50)	94.1	0.00	
	Age ≥ 75	8	42,366	1.18 (1.08–1.28)	87.1	0.00	
Population	General	50	197,042	1.36 (1.27–1.46)	90.9	0.00	0.00
	CVD	6	5965	1.36 (1.12–1.64)	48.4	0.00	
	T2DM	3	10,685	1.38 (1.09–1.75)	69.4	0.01	
	Cancer: Present vs. Absent	1	19,360	1.33 (1.20–1.48)	NA	0.00	
Instrument used	Validated social network indexes	30	122,050	1.44 (1.30–1.60)	89.2	0.00	0.06
	Other	31	111,229	1.28 (1.21–1.36)	82.2	0.00	
Country	Europe	20	31,589	1.25 (1.12–1.39)	87.7	0.00	0.00
	North America	21	81,049	1.51 (1.37–1.67)	61.8	0.00	
	Other*	20	120,641	1.24 (1.20–1.29)	38.1	0.00	
Follow-up	< 5 years	18	89,115	1.46 (1.24–1.71)	96.8	0.00	0.01
	≥ 5 years	43	144,164	1.31 (1.24–1.39)	79.7	0.00	
NOS scale score	≥ 7	53	191,800	1.31 (1.24–1.38)	87.7	0.00	0.04
	≤ 6	8	41,479	1.81 (1.34–2.46)	83.3	0.00	
Adjusted for income	Yes	14	56,245	1.44 (1.27–1.63)	78.4	0.00	0.21
	No	47	177,034	1.32 (1.23–1.40)	89.9	0.00	
Adjusted for mental symptoms	Yes	31	141,676	1.35 (1.24–1.47)	94.3	0.00	0.97
	No	30	91,603	1.35 (1.25–1.47)	69.2	0.00	
Adjusted for physical symptoms	Yes	49	184,012	1.37 (1.28–1.47)	91.6	0.00	0.13
	No	12	49,267	1.24 (1.11–1.39)	57.2	0.00	
Adjusted for cognitive function	Yes	6	25,868	1.28 (1.06–1.54)	87.9	0.00	0.55
	No	55	207,411	1.36 (1.28–1.45)	87.8	0.00	

* The ‘Other’ region category includes countries outside Europe and North America, such as China, Japan, Korea, and Mexico. Notes: CVD = cardiovascular disease, HR = Hazzard ratio, I^2^ = heterogeneity, NA = not applicable, NOS = Newcastle Ottawa Scale, P = p-value, T2DM = type 2 diabetes mellitus. Group differences were calculated using Cochran’s Q test for subgroup differences

In the association between living alone and all-cause mortality (Table 3), males exhibited a higher HR of 1.49 (95% CI: 1.21–1.84) than females. For individuals aged 65 years and above, the HR was 1.29 (95% CI: 1.18–1.42), which was significantly higher than in age groups of ≥ 50 or ≥ 75 years. Geographically, the Other Regions category had the highest HR at 1.29 (95% CI: 1.09–1.52) than Europe and North America.

**Table 3:** Summary HRs in various subgroup analyses for the association between living alone and all-cause mortality

Category	Subgroup	No. of studies/ estimates	Sample size	HR (95% CI) random effects	I^2^ (%)	*P*	Group Differences (Cochran’s Q test *P*)
Sex	Both	13	43,341	1.18 (1.11–1.26)	59.9	0.00	0.04
	Male	8	18,015	1.49 (1.21–1.84)	52.8	0.00	
	Female	8	28,309	1.07 (0.91–1.26)	45.2	0.40	
Age	Age ≥ 50–65	9	28,658	1.10 (1.02–1.19)	30.0	0.02	0.01
	Age ≥ 65	20	76,795	1.29 (1.18–1.42)	71.2	0.00	
	Age ≥ 75	3	1078	1.41 (0.74–2.68)	0.00	0.30	
Population	General	27	51,716	1.18 (1.11–1.26)	52.9	0.00	0.24
	Hip fracture	2	34,837	1.39 (1.07–1.78)	92.7	0.01	
Instrument used	Validated questionnaires	0	NA	NA	NA	NA	NA
	Other	29	86,553	1.21 (1.13–1.30)	69.1	0.00	
Country	Europe	15	53,850	1.23 (1.11–1.37)	72.1	0.00	0.03
	North America	4	18,942	1.08 (1.03–1.14)	0.00	0.00	
	Other*	10	13,761	1.29 (1.09–1.52)	56.1	0.00	
Follow-up	< 5 years	7	6716	1.05 (0.89–1.24)	26.9	0.58	0.06
	≥ 5 years	22	79,837	1.24 (1.16–1.34)	68.6	0.00	
NOS scale score	≥ 7	28	83,399	1.22 (1.13–1.31)	71.1	0.00	0.53
	≤ 6	1	3154	1.14 (0.95–1.37)	NA	0.17	
Adjusted for income	Yes	9	27,671	1.11 (1.07–1.15)	0.01	0.00	0.03
	No	20	58,882	1.26 (1.13–1.41)	68.2	0.00	
Adjusted for mental symptoms	Yes	8	21,122	1.23 (1.12–1.35)	16.9	0.00	0.83
	No	21	65,431	1.21 (1.11–1.32)	77.2	0.00	
Adjusted for physical symptoms	Yes	17	32,185	1.21 (1.13–1.29)	23.5	0.00	0.82
	No	12	54,368	1.19 (1.05–1.35)	85.4	0.01	
Adjusted for cognitive function	Yes	5	13,399	1.18 (1.03–1.36)	59.3	0.02	0.69
	No	24	73,154	1.22 (1.12–1.33)	71.3	0.00	

* The ‘Other’ region category includes countries outside Europe and North America, such as Australia, China, Japan, and Singapore. Notes: CVD = cardiovascular disease, HR = Hazzard ratio, I^2^ = heterogeneity, NA = not applicable, NOS = Newcastle Ottawa Scale, P = p-value, T2DM = type 2 diabetes mellitus. Group differences were calculated using Cochran’s Q test for subgroup differences

### Meta-regression analysis

The meta-regression analysis for the association between loneliness and all-cause mortality found that only longer follow-up periods (> 5 years) were associated with smaller effect sizes (*p* = 0.02; supplementary figure S19). The model, however, did not explain a significant portion of the heterogeneity observed in the effect sizes (R^2^ = 0.01%).

Meta-regression analysis for the association between social isolation and all-cause mortality found that validated social network indexes was associated with larger effect sizes (*p* = 0.02), while studies of higher quality (NOS ≥ 7) resulted in smaller effect sizes (*p* = 0.01). The model explained a moderate portion of the variability in effect sizes (R^2^ = 17.0%).

Meta-regression analysis for the association between living alone and all-cause mortality found that female participants (*p* = 0.00) and income-adjusted studies (*p* = 0.03) reported smaller effect sizes, while longer follow-up periods (> 5 years, *p* = 0.05) were associated with larger effect sizes (supplementary figure S21). The model explained a substantial portion of the variability in effect sizes (R^2^ = 61.4%).

## Discussion

### Principal findings

In this systematic review with meta-analysis, we included a total of 86 prospective cohort or longitudinal studies, with a focus on the general older adult population as well as specific subpopulations, such as those diagnosed with CVD, cancer, T2DM, and hip fractures. Our meta-analytic procedures found that loneliness, social isolation, and living alone are each significantly associated with increased risk of all-cause mortality in older adults. The HRs for these associations vary (from 1.35 to 1.14), with social isolation having the strongest association, followed by living alone and loneliness. Additionally, our analysis indicated a noteworthy association between each of these social factors—loneliness, living alone, and social isolation—and an increased risk of CVD mortality, highlighting the impact of these social factors on cardiovascular health. Contrarywise, no substantial evidence was found to associate loneliness, social isolation, and living alone with cancer mortality. This finding features the complexity of the associations between social factors and specific mortality outcomes, warranting further in-depth investigation. However, there was substantial heterogeneity in these associations, with variations based on factors such as sex, age, geographical region, chronic diseases, and study quality. Meta-regression analysis identified factors like longer follow-up periods, female sex, validated social network indices, adjustments for cognitive function, and study quality as significant predictors of mortality risks. In sum, our findings are congruent with a substantial corpus of evidence highlighting the detrimental effects of loneliness, social isolation, and living alone on all-cause and CVD mortality [6, 7, 17, 18, 22, 37]. Consequently, our study accentuates the crucial need for public health strategies to address this triad of social factors to ameliorate health outcomes in the aging demographic [134], though further investigation is warranted due to the variability and heterogeneity observed in these studies [3, 18, 135].

### Comparison with previous work

Specifically, our findings indicate that social isolation was associated with a 35% increased likelihood of all-cause mortality in older adults, followed by living alone with a 21% increase, and loneliness with a 14% increase. These figures align closely with other meta-analytical findings. For instance, Holt-Lunstad et al. [7] found that social isolation was associated with an average increased likelihood of mortality of around 26%. Similarly, two other meta-analyses reported pooled effect sizes for social isolation, indicating a 32% [37] and 33% [39] increased risk of all-cause mortality, respectively, which aligns with our study’s findings. Furthermore, our study also demonstrated an elevated risk of CVD mortality linked to social isolation, a trend consistent with the findings of the latter study [37]. However, unlike their results [37], we did not confirm a significant increase in the likelihood of cancer mortality associated with social isolation. These differences may be due to several factors, including differences in the age composition of the study populations as well as differences in the definition and measurement of social isolation and specific types of mortality between studies, which could also account for differences in results. When it comes to living alone, previous work has also reported similar numbers, ranging from 15 to 32% increased risk of mortality [6, 7, 22]. In terms of loneliness, Zhou et al. [18], Schutter et al. [17] and Rico-Uribe et al. [38] noted a 9%, 10% and 22% increased hazard of all-cause mortality associated with loneliness in older adults, respectively. These percentages are slightly lower than those in our study. Additionally, Wang et al. [37] found loneliness to be significantly linked to a 14% higher risk of all-cause mortality and a 9% increase in cancer mortality among adults. Holt-Lunstad et al. [7] reported a 26% increased risk of mortality due to loneliness; however, both of these latter studies [7, 126] did not exclusively focus on older adults. Overall, these results provide consistent evidence of the increased mortality risk associated with loneliness, social isolation, and living alone, although there may be slight variations in the reported effect sizes. Furthermore, the stronger association with increased all-cause mortality in older adults compared to the impacts of living alone or loneliness may potentially be explained by multifaceted impacts of social isolation in terms of psychological, physical, cognitive, and health-related aspects [5, 10, 135, 136]. While living alone and loneliness are significant factors, they do not necessarily imply a complete absence of social interaction or support. One can live alone but still maintain an active social life and have strong social support networks [137]. Similarly, loneliness is a subjective feeling and may not always be related to the actual level of social interaction and support [7, 10, 15]. It is also possible that social connections may encourage healthier behaviours through advice and support, while social isolation could lead to a lack of motivation for health maintenance, possibly resulting in unhealthy behaviour [136]. The observed associations with CVD mortality, cancer mortality, and other mortality types, however, warrant further investigation with a focus on understanding the underlying mechanisms [138].

Our subgroup analyses revealed some important findings. Individuals with CVD showed a doubled risk of mortality due to loneliness, while the choice of loneliness measurement method and follow-up duration impacted risk estimates. Adjusting for cognitive function moderated the association, indicating its confounding role in the loneliness-mortality association [139, 140]. Similarly, cancer patients faced significantly higher mortality risks, reflecting the potential dangers of social isolation in this group [37]. Study quality variations underscored the importance of methodological rigor. Geographical differences were notable, with North America showing a distinct impact of social isolation on mortality. These regional differences, particularly in North America, have been consistently documented in the literature [18, 136]. Cultural factors such as social norms, family structures, and community practices play a significant role in shaping how loneliness and social isolation are defined and experienced worldwide. For instance, in collectivist societies, extended family support may interact differently with social isolation compared to individualistic cultures [141, 142]. In studying living alone and all-cause mortality, sex disparities emerged, with men facing higher risks, as previously reported [6]. Older adults over 65 years were notably vulnerable, with a significantly increased risk of mortality, a finding also supported by previous work [6]. However, the lack of significant findings in the ≥ 75 age group may reflect survival bias or insufficient statistical power due to the smaller sample size [143]. Further research with larger cohorts is necessary to clarify the risk in this age group and investigate potential protective factors, such as post-retirement role shifts or enhanced resilience.

The meta-regression analysis also showed intriguing patterns in the association between these social dynamics and all-cause mortality. Particularly, for the association between loneliness and mortality, longer follow-up periods appeared to yield diminished effect sizes, suggesting a potential attenuation of the association over time. Regarding social isolation, the use of validated social network indexes was associated with larger effect sizes, indicating a potentially stronger impact on mortality when more comprehensive measures of social isolation were employed. Contrarywise, studies of higher quality, reported smaller effect sizes, underscoring the importance of methodological rigor in assessing this association. In the analysis of living alone and all-cause mortality, several noteworthy trends emerged. Female participants and income-adjusted studies reported smaller effect sizes, suggesting potential mitigating factors influencing the association in these subgroups. Conversely, longer follow-up periods were associated with larger effect sizes, implying a cumulative impact of living alone on mortality over time. The discrepancy observed with follow-up periods may reflect the differing natures of loneliness and living alone. We can speculate that loneliness, a dynamic experience, may diminish as individuals adapt or improve their circumstances, while living alone, a stable condition, carries risks that accumulate with age. Loneliness likely impacts mortality through immediate psychological pathways, such as distress or behavioral changes, whereas living alone involves structural risks, like reduced support or emergency care, that worsen over time with age and frailty. These findings highlight the intricate interplay between individual characteristics, study methodologies, and temporal dynamics in shaping the association between social dynamics and mortality outcomes [1, 3–5].

### Possible mechanisms and explanations

Despite the exact mechanisms underlying the association between loneliness, social isolation, and living alone and increased mortality risk not being fully understood [138], the literature indicates that this triad of social dynamics is closely linked to premature death through various interconnected pathways [10]. Initially, such social dynamics can engender an absence of communal support and are influenced by life transitions and changes in health status [137], thereby intensifying sensations of distress, despondency, and apprehension, particularly in older adults. Devoid of the emotional nourishment and pragmatic aid proffered by social ties, individuals may endure adverse effects on their psychological well-being, culminating in a heightened risk of mortality [10, 119, 137]. Furthermore, they frequently coincide with detrimental habits, including tobacco usage, immoderate alcohol intake, and suboptimal dietary choices. These practices foster the emergence of chronic ailments such as cardiovascular diseases, neoplastic disorders, and pulmonary conditions, each associated with elevated mortality rates [3, 21, 136, 144]. Additionally, social isolation and living alone are correlated with alterations in immune functionality, encompassing diminished responses to infections and increased inflammatory activity [3, 23, 24, 136]. Persistent inflammation is linked to a spectrum of health complications, encompassing cardiovascular disorders, autoimmune diseases, and cancer, all of which amplify mortality risk [24, 145]. Moreover, social isolation is tied to augmented levels of cardiovascular risk factors like hypertension, obesity, and dyslipidemia. These elements precipitate the onset of cardiovascular maladies such as myocardial infarctions and cerebral strokes, principal causes of death globally [1, 10, 15]. Living alone also poses challenges in accessing healthcare provisions, including transportation for medical consultations, and managing healthcare logistics [6, 137]. Moreover, individuals living alone may encounter difficulties in adhering to medication regimes and maintaining self-care practices, resulting in unmanaged health conditions and an increased risk of mortality [137, 146]. Additionally, the stress associated with living alone, particularly in the twilight years, can exert detrimental impacts on both mental and physical health [137]. The experience of loneliness, apprehensions regarding safety and security, and anxieties about aging can lead to persistent stress, which relates to deleterious health outcomes and an amplified risk of mortality [137].

### Strength and limitations

Our study signifies a novel undertaking encircling a range of pertinent social dynamics, including loneliness, social isolation, living alone, and various mortality outcomes, thereby providing a comprehensive overview of these associations, particularly among older adults by integrating data sourced from a diverse array of countries worldwide. Furthermore, the prevalence of high-quality studies advocates a sturdy methodological underpinning for the drawn conclusions, enhancing the robustness of our findings. However, the presence of moderate-quality studies stresses the importance of carefully considering potential methodological limitations when interpreting the overall results. Additionally, our analytical approaches bolster the robustness of our findings by thoroughly examining heterogeneity, cumulative evidence, stability of the findings, and potential moderating factors.

Moving to potential limitations, it’s crucial to address the constraints arising from the language and publication selection. The exclusion of non-English studies could lead to language bias, potentially omitting significant data from diverse geographical regions. This exclusion likely limited our ability to capture insights from countries where loneliness, social isolation, and living alone are experienced and interpreted differently due to cultural, linguistic, or societal factors. Future studies should aim to include research published in multiple languages to create a more inclusive and representative understanding of these complex social constructs. Additionally, reliance on published studies could contribute to publication bias, as unpublished data might offer alternative perspectives [147]. While our findings highlight geographical variations, the prevalence of studies concentrated in Europe and North America restricts the generalizability of results to other regions. Future research should prioritize including studies from underrepresented regions such as Asia, Africa, and South America, ensuring a more globally relevant perspective. Moreover, future investigations could examine how cultural dimensions and regional differences contribute to variations in health outcomes associated with loneliness, social isolation, and living alone.

Another notable limitation is the lack of standardized metrics for measuring loneliness across the included studies. The instruments used varied widely, ranging from validated multi-item scales (e.g., UCLA Loneliness Scale, De Jong Gierveld Loneliness Scale) to single-item measures. This variability, combined with a reliance on self-reported data, introduces heterogeneity that complicates comparability across studies. Although our meta-regression partially addressed this issue by identifying predictors such as the use of validated scales, unexplained variability persists. Single-item measures, in particular, may overestimate the effects of loneliness on mortality due to their simplicity and potential for misclassification. These measures often fail to capture the multidimensional nature of loneliness, which includes both emotional and social components. As a result, they may lead to an overgeneralization of loneliness’s effects. This underscores the critical need for standardized, validated tools to measure loneliness and related constructs. Such tools would improve consistency across future studies and meta-analyses, ultimately strengthening the evidence base in this field. Additionally, the exclusion criteria based on study design or participant age may limit the scope and applicability of our findings. Variability in data reporting among studies also affects the consistency and comparability of our meta-analysis results, adding complexity to their interpretation. While we relied on prospective data, we cannot conclusively establish causality in the observed relationships. Furthermore, the limited number of studies addressing certain outcomes and the lack of significant findings in specific areas highlight gaps requiring further exploration.

Finally, this study focused on examining the independent effects of loneliness, social isolation, and living alone on health and well-being outcomes for older adults, without addressing their potential combined or synergistic effects. While these constructs are distinct, they may overlap in complex ways that could amplify their impact on health outcomes. Future research should investigate these interactions to better understand how loneliness, social isolation, and living alone might collectively influence mortality risks. Additionally, we did not analyse the role of broader contextual factors, such as societal changes, cultural norms, or technological advancements, which may shape these relationships. Exploring these mechanisms, as well as the influence of specific health conditions and demographic subgroups, could provide deeper insights and inform more targeted interventions.

### Implications for practice

According to our findings, the establishment and execution of community-centered initiatives are pivotal in mitigating loneliness and social isolation among older adults [1, 12, 138]. Initiatives like social clubs, volunteer programs, regular community events, and befriending services [148] can significantly contribute to this effort. In transitioning from communal undertakings to healthcare settings, it becomes imperative to advocate healthcare professionals to incorporate screenings for loneliness and social isolation into their routine health evaluations. This practice, particularly aimed at older adults, is instrumental in identifying individuals at risk and ensuring they receive prompt and appropriate support [1, 149]. Moreover, the significance of policymakers in this context is undeniable. It is essential that they carefully consider the ramifications of loneliness and social isolation on health while formulating policies for the older adult demographic [1], as interventions need to be tailored to individual or group needs and there’s no one-size-fits-all solution [138]. This strategy ought to be augmented by continued research aimed at investigating and crafting efficacious interventions to address these critical social determinants [138]. Finally, legislative measures and policy frameworks aimed at tackling marginalization and discrimination against ageism can play a pivotal role in nurturing stronger social connections [150].

## Conclusions

In essence, our meta-analysis confirmed that loneliness, social isolation, and living alone are associated with an increased risk of mortality in older adults, particularly in terms of all-cause and CVD mortality. These findings underscore the significance of addressing these social factors as potential public health concerns. Nonetheless, the variability in effect sizes and the presence of heterogeneity and publication bias in some analyses suggest the need for further research to comprehensively understand these associations. Moreover, investigating the cumulative effect of these factors on mortality risks is of considerable interest. By examining how the combined influence of loneliness, social isolation, and living alone contributes to mortality outcomes over time, we may gain deeper insights into the complex interplay of these social dynamics on health and longevity in older adults. Such analysis not only elucidates the additive effects of these factors but also offers valuable insights for developing targeted interventions aimed at mitigating the adverse health consequences associated with this triad of social dynamics in this vulnerable population.

## Electronic supplementary material

Below is the link to the electronic supplementary material.

Supplementary Material 1

## Data Availability

No datasets were generated or analysed during the current study.
