# Supplementary material for: Loneliness, social isolation, and living alone: a comprehensive systematic review, meta-analysis, and meta-regression of mortality risks in older adults
Source: Aging Clin Exp Res. 2025 Jan 21;37(1):29. doi: 10.1007/s40520-024-02925-1 (PMC11750934; doi:10.1007/s40520-024-02925-1)
Supplement: Supplementary file 1 — Supplementary Material 1 [file 40520_2024_2925_MOESM1_ESM.docx]

**Supplementary Material**

**Loneliness, Social Isolation, and Living Alone: A Comprehensive Systematic Review, Meta-Analysis and Meta-Regression of Mortality Risks in Older Adults**

[Search Strategy 3](#_Toc160569337)

[PubMed Search Strategy 3](#_Toc160569338)

[APA PsycINFO and CINAHL (via EBSCOhost ) Search Strategy 3](#_Toc160569339)

[PRISMA Checklist and PRISMA 2020 for Abstracts Checklist 1](#_Toc160569340)

[MOOSE Checklist for Reporting of Meta-analyses of Observation Studies 6](#_Toc160569341)

[Table S1. Excluded studies with reasons 8](#_Toc160569342)

[Table S2. Characteristics of included studies for the association between loneliness and mortality 11](#_Toc160569343)

[Table S3. Characteristics of included studies for the association between social isolation and mortality 22](#_Toc160569344)

[Table S4. Characteristics of included studies for the association between living alone and mortality 31](#_Toc160569345)

[Table S5. New-castle-Ottawa scores for the included studies 35](#_Toc160569346)

[Figure S1. Funnel plot of publications bias (p=0.0012) for the association between loneliness and all-cause mortality. 38](#_Toc160569347)

[Figure S2. Cumulative meta-analysis for the association between loneliness and all-cause mortality. 39](#_Toc160569348)

[Figure S3. Leave-one-out sensitivity meta-analysis for the association between loneliness and all-cause mortality. 40](#_Toc160569349)

[Figure S4. Forest plot for the association between loneliness and CVD and cancer mortality Notes: F=female, M=male 41](#_Toc160569350)

[Figure S5. Cumulative meta-analysis for the association between loneliness and CVD mortality. 42](#_Toc160569351)

[Figure S6. Leave-one-out sensitivity meta-analysis for the association between loneliness and CVD mortality. 43](#_Toc160569352)

[Figure S7. Funnel plot of publications bias (p=0.000) for the association between social isolation and all-cause mortality. 44](#_Toc160569353)

[Figure S8. Cumulative meta-analysis for the association between social isolation and all-cause mortality. 45](#_Toc160569354)

[Figure S9. Leave-one-out sensitivity meta-analysis for the association between social isolation and all-cause mortality. 46](#_Toc160569355)

[Figure S10. Forest plot for the association between social isolation and CVD, cancer, and other mortality Notes: F=female, M=male 47](#_Toc160569356)

[Figure S11. Cumulative meta-analysis for the association between social isolation and CVD mortality. 48](#_Toc160569357)

[Figure S12. Leave-one-out sensitivity meta-analysis for the association between social isolation and CVD mortality. Notes: F=female, M=male 48](#_Toc160569358)

[Figure S13. Cumulative meta-analysis for the association between social isolation and cancer mortality. Notes: F=female, M=male 49](#_Toc160569359)

[Figure S15. Funnel plot of publications bias (p=0.4296) for the association between living alone and all-cause mortality. 50](#_Toc160569360)

[Figure S16. Cumulative meta-analysis for the association between living alone and all-cause mortality. Notes: F=female, M=male 51](#_Toc160569361)

[Figure S17. Leave-one-out sensitivity meta-analysis for the association between living alone and all-cause mortality. Notes: F=female, M=male 52](#_Toc160569362)

[Figure S18. Forest plot for the association between living alone and CVD, cancer, and other mortality 53](#_Toc160569363)

[Figure S19. Bubble plot for follow-up periods regarding the association between loneliness and all-cause mortality. 54](#_Toc160569364)

[Figure S20. Bubble plot for validated social network indexes, and NOS scale regarding the association between social isolation and all-cause mortality. 55](#_Toc160569365)

[Figure S21. Bubble plot for female (sex), adjusted for income, and follow-up periods regarding the association between living alone and all-cause mortality. 56](#_Toc160569366)

## Search Strategy

## PubMed Search Strategy

Search: (loneliness OR lonel* OR social isolation OR social isolat* OR living alone OR live alone OR social alienat* OR social network* OR solitud* OR social relationships OR social participation OR social integration OR social exclusion) AND (mortality OR all-cause mortality OR cause-specific mortality OR mortality rate OR death rate OR longevity OR proportional hazard models OR dead OR death OR dying OR surviv* OR cause of death OR decease* OR premature mortality) AND (older adults OR elderly OR elder* OR geriatric OR aged population OR seniors) AND (cohort study OR prospective cohort OR longitudinal) Filters: Humans

Results: 6,155

## APA PsycINFO and CINAHL (via EBSCOhost ) Search Strategy

(loneliness or lonel* or social isolation or social isolat* or living alone or live alone or social alienat* or social network* or solitud* or social relationships or social participation or social integration or social exclusion) ) and (mortality or all-cause mortality or cause-specific mortality or mortality rate or death rate or longevity or proportional hazard models or dead or death or dying or surviv* or cause of death or decease* or premature mortality ) and (older adults or elderly or elder* or geriatric or aged population or seniors ) and (cohort study or prospective cohort or longitudinal ) Population Group: Human AND Apply related words; Also search within the full text of the articles; Apply equivalent subjects

Results: 5,807

## PRISMA Checklist and PRISMA 2020 for Abstracts Checklist

| **Section and Topic** | **Item #** | **Checklist item** | **Location where item is reported** |
| --- | --- | --- | --- |
| **TITLE** | | |  |
| Title | 1 | Identify the report as a systematic review. | Page 1 |
| **ABSTRACT** | | |  |
| Abstract | 2 | See the PRISMA 2020 for Abstracts checklist. | Page 6 supplementary material |
| **INTRODUCTION** | | |  |
| Rationale | 3 | Describe the rationale for the review in the context of existing knowledge. | Page 4-5 |
| Objectives | 4 | Provide an explicit statement of the objective(s) or question(s) the review addresses. | Page 5 |
| **METHODS** | | |  |
| Eligibility criteria | 5 | Specify the inclusion and exclusion criteria for the review and how studies were grouped for the syntheses. | Page 7-8 |
| Information sources | 6 | Specify all databases, registers, websites, organisations, reference lists and other sources searched or consulted to identify studies. Specify the date when each source was last searched or consulted. | Page 6-8 |
| Search strategy | 7 | Present the full search strategies for all databases, registers and websites, including any filters and limits used. | Page 3 supplementary material |
| Selection process | 8 | Specify the methods used to decide whether a study met the inclusion criteria of the review, including how many reviewers screened each record and each report retrieved, whether they worked independently, and if applicable, details of automation tools used in the process. | Pages 7-8 |
| Data collection process | 9 | Specify the methods used to collect data from reports, including how many reviewers collected data from each report, whether they worked independently, any processes for obtaining or confirming data from study investigators, and if applicable, details of automation tools used in the process. | Pages 7-8 |
| Data items | 10a | List and define all outcomes for which data were sought. Specify whether all results that were compatible with each outcome domain in each study were sought (e.g. for all measures, time points, analyses), and if not, the methods used to decide which results to collect. | Page 7 |
|  | 10b | List and define all other variables for which data were sought (e.g. participant and intervention characteristics, funding sources). Describe any assumptions made about any missing or unclear information. | Page 8 |
| Study risk of bias assessment | 11 | Specify the methods used to assess risk of bias in the included studies, including details of the tool(s) used, how many reviewers assessed each study and whether they worked independently, and if applicable, details of automation tools used in the process. | Page 9 |
| Effect measures | 12 | Specify for each outcome the effect measure(s) (e.g. risk ratio, mean difference) used in the synthesis or presentation of results. | Pages 8-9 |
| Synthesis methods | 13a | Describe the processes used to decide which studies were eligible for each synthesis (e.g. tabulating the study intervention characteristics and comparing against the planned groups for each synthesis (item #5)). | Page 8 |
|  | 13b | Describe any methods required to prepare the data for presentation or synthesis, such as handling of missing summary statistics, or data conversions. | NA |
|  | 13c | Describe any methods used to tabulate or visually display results of individual studies and syntheses. | Pages 9-10 |
|  | 13d | Describe any methods used to synthesize results and provide a rationale for the choice(s). If meta-analysis was performed, describe the model(s), method(s) to identify the presence and extent of statistical heterogeneity, and software package(s) used. | Pages 9-10 |
|  | 13e | Describe any methods used to explore possible causes of heterogeneity among study results (e.g. subgroup analysis, meta-regression). | Page 10 |
|  | 13f | Describe any sensitivity analyses conducted to assess robustness of the synthesized results. | Page 10 |
| Reporting bias assessment | 14 | Describe any methods used to assess risk of bias due to missing results in a synthesis (arising from reporting biases). | Page 10 |
| Certainty assessment | 15 | Describe any methods used to assess certainty (or confidence) in the body of evidence for an outcome. | NA |
| **RESULTS** | | |  |
| Study selection | 16a | Describe the results of the search and selection process, from the number of records identified in the search to the number of studies included in the review, ideally using a flow diagram. | Page 11 |
|  | 16b | Cite studies that might appear to meet the inclusion criteria, but which were excluded, and explain why they were excluded. | Page 11, supplementary material (Table S1) |
| Study characteristics | 17 | Cite each included study and present its characteristics. | Page 11, Tables S2, S3, and S4 in the supplementary material. |
| Risk of bias in studies | 18 | Present assessments of risk of bias for each included study. | Page 12, moderate (Table S5 in the supplementary material). |
| Results of individual studies | 19 | For all outcomes, present, for each study: (a) summary statistics for each group (where appropriate) and (b) an effect estimate and its precision (e.g. confidence/credible interval), ideally using structured tables or plots. |  |
| Results of syntheses | 20a | For each synthesis, briefly summarise the characteristics and risk of bias among contributing studies. | Pages 12-18 |
|  | 20b | Present results of all statistical syntheses conducted. If meta-analysis was done, present for each the summary estimate and its precision (e.g. confidence/credible interval) and measures of statistical heterogeneity. If comparing groups, describe the direction of the effect. | Pages 12-18 |
|  | 20c | Present results of all investigations of possible causes of heterogeneity among study results. | Pages 12-18 |
|  | 20d | Present results of all sensitivity analyses conducted to assess the robustness of the synthesized results. | Page 18 |
| Reporting biases | 21 | Present assessments of risk of bias due to missing results (arising from reporting biases) for each synthesis assessed. | Page 18 |
| Certainty of evidence | 22 | Present assessments of certainty (or confidence) in the body of evidence for each outcome assessed. | NA |
| **DISCUSSION** | | |  |
| Discussion | 23a | Provide a general interpretation of the results in the context of other evidence. | Page 19 |
|  | 23b | Discuss any limitations of the evidence included in the review. | Page 25 |
|  | 23c | Discuss any limitations of the review processes used. | Page 25 |
|  | 23d | Discuss implications of the results for practice, policy, and future research. | Page 26 |
| **OTHER INFORMATION** | | |  |
| Registration and protocol | 24a | Provide registration information for the review, including register name and registration number, or state that the review was not registered. | Page 6 |
|  | 24b | Indicate where the review protocol can be accessed, or state that a protocol was not prepared. | Page 6 |
|  | 24c | Describe and explain any amendments to information provided at registration or in the protocol. | Pages 10-11 |
| Support | 25 | Describe sources of financial or non-financial support for the review, and the role of the funders or sponsors in the review. | Page 27 |
| Competing interests | 26 | Declare any competing interests of review authors. | Page 27 |
| Availability of data, code and other materials | 27 | Report which of the following are publicly available and where they can be found: template data collection forms; data extracted from included studies; data used for all analyses; analytic code; any other materials used in the review. | Supplementary material |

| **Section and Topic** | **Item #** | **Checklist item** | **Reported (Yes/No)** |
| --- | --- | --- | --- |
| **TITLE** | | |  |
| Title | 1 | Identify the report as a systematic review. | Yes |
| **BACKGROUND** | | |  |
| Objectives | 2 | Provide an explicit statement of the main objective(s) or question(s) the review addresses. | Yes |
| **METHODS** | | |  |
| Eligibility criteria | 3 | Specify the inclusion and exclusion criteria for the review. | Yes |
| Information sources | 4 | Specify the information sources (e.g. databases, registers) used to identify studies and the date when each was last searched. | Yes |
| Risk of bias | 5 | Specify the methods used to assess risk of bias in the included studies. | Yes |
| Synthesis of results | 6 | Specify the methods used to present and synthesise results. | Yes |
| **RESULTS** | | |  |
| Included studies | 7 | Give the total number of included studies and participants and summarise relevant characteristics of studies. | Yes |
| Synthesis of results | 8 | Present results for main outcomes, preferably indicating the number of included studies and participants for each. If meta-analysis was done, report the summary estimate and confidence/credible interval. If comparing groups, indicate the direction of the effect (i.e. which group is favoured). | Yes |
| **DISCUSSION** | | |  |
| Limitations of evidence | 9 | Provide a brief summary of the limitations of the evidence included in the review (e.g. study risk of bias, inconsistency and imprecision). | Yes |
| Interpretation | 10 | Provide a general interpretation of the results and important implications. | Yes |
| **OTHER** | | |  |
| Funding | 11 | Specify the primary source of funding for the review. | Yes |
| Registration | 12 | Provide the register name and registration number. | Yes |

From: Page MJ, McKenzie JE, Bossuyt PM, Boutron I, Hoffmann TC, Mulrow CD, et al. The PRISMA 2020 statement: an updated guideline for reporting systematic reviews. BMJ 2021;372:n71. doi: 10.1136/bmj.n71

## MOOSE Checklist for Reporting of Meta-analyses of Observation Studies

| **Item No** | **Recommendation** | **Reported on Page No** |
| --- | --- | --- |
| Reporting of background should include | | |
| 1 | Problem definition | 4 |
| 2 | Hypothesis statement | 4 |
| 3 | Description of study outcome(s) | 4-5 |
| 4 | Type of exposure or intervention used | 4-5 |
| 5 | Type of study designs used | 4-5 |
| 6 | Study population | 4-5 |
| Reporting of search strategy should include | | |
| 7 | Qualifications of searchers (eg, librarians and investigators) | Title page |
| 8 | Search strategy, including time period included in the synthesis and key words | 6, Supplementary Material (see the 'Search Strategy' section). |
| 9 | Effort to include all available studies, including contact with authors | NA |
| 10 | Databases and registries searched | 6 |
| 11 | Search software used, name and version, including special features used (eg, explosion) | NA |
| 12 | Use of hand searching (eg, reference lists of obtained articles) | 6 |
| 13 | List of citations located and those excluded, including justification | supplementary material (Table S1). |
| 14 | Method of addressing articles published in languages other than English | 8 |
| 15 | Method of handling abstracts and unpublished studies | NA |
| 16 | Description of any contact with authors | NA |
| Reporting of methods should include | | |
| 17 | Description of relevance or appropriateness of studies assembled for assessing the hypothesis to be tested | 7-8 |
| 18 | Rationale for the selection and coding of data (eg, sound clinical principles or convenience) | 7-8 |
| 19 | Documentation of how data were classified and coded (eg, multiple raters, blinding and interrater reliability) | 7-8 |
| 20 | Assessment of confounding (eg, comparability of cases and controls in studies  where appropriate) | 9 |
| 21 | Assessment of study quality, including blinding of quality assessors, stratification or regression on possible predictors of study results | 9-10 |
| 22 | Assessment of heterogeneity | 9-10 |
| 23 | Description of statistical methods (eg, complete description of fixed or random effects models, justification of whether the chosen models account for predictors of study results, dose-response models, or cumulative meta-analysis) in sufficient detail to be replicated | 9-10 |
| 24 | Provision of appropriate tables and graphics | Tables and figures both in main text and supplementary material |
| Reporting of results should include | | |
| 25 | Graphic summarizing individual study estimates and overall estimate | Figure 2,3,4 |
| 26 | Table giving descriptive information for each study included | Tables S2, S3, and S4 in the supplementary material |
| 27 | Results of sensitivity testing (eg, subgroup analysis) | Tables 1,2,3 |
| 28 | Indication of statistical uncertainty of findings | NA |
| Reporting of discussion should include | | |
| 29 | Quantitative assessment of bias (eg, publication bias) | 12 |
| 30 | Justification for exclusion (eg, exclusion of non-English language citations) | 8 |
| 31 | Assessment of quality of included studies | Table S5 in the supplementary material). |
| Reporting of conclusions should include | | |
| 32 | Consideration of alternative explanations for observed results | 19-20 |
| 33 | Generalization of the conclusions (ie, appropriate for the data presented and within the domain of the literature review) | 24-25 |
| 34 | Guidelines for future research | 26 |
| 35 | Disclosure of funding source | 27 |

*From*: Stroup DF, Berlin JA, Morton SC, et al, for the Meta-analysis Of Observational Studies in Epidemiology (MOOSE) Group. Meta-analysis of Observational Studies in Epidemiology. A Proposal for Reporting. *JAMA*. 2000;283(15):2008-2012. doi: 10.1001/jama.283.1

| Table S1. Excluded studies with reasons | Reason |
| --- | --- |
| Alcaraz, K. I. et al. Social isolation and mortality in US Black and white men and women. Am. J. Epidemiol. 188, 102–109 (2019). | Age <50 |
| Ayalon L, Shiovitz-Ezra S. The relationship between loneliness and passive death wishes in the second half of life. Int Psychogeriatr. 2011;23(10):1677-1685. doi:10.1017/S1041610211001384 | No outcome of interest |
| Beasley, J. M. et al. Social networks and survival after breast cancer diagnosis. J. Cancer Surviv. 4, 372–380 (2010). | Age range not reported |
| Beller, J. & Wagner, A. Loneliness, social isolation, their synergistic interaction, and mortality. Health Psychol. 37, 808–813 (2018). | Age <50 |
| Beller, J. Loneliness and mortality: the moderating effect of positive affect. Appl. Psychol. Health Well Being. 15(1), 49-65 (2023). | Age <50 |
| Berkman, L. F. et al. Social integration and mortality: a prospective study of French employees of Electricity of France–Gas of France: the GAZEL Cohort. Am J Epidemiol. 159(2), 167–174 (2004). | Age <50 |
| Denollet J, Maas K, Knottnerus A, Keyzer JJ, Pop VJ. Anxiety predicted premature all-cause and cardiovascular death in a 10-year follow-up of middle-aged women. J Clin Epidemiol. 2009;62(4):452–456. doi: 10.1016/j.jclinepi.2008.08.006 | Age <50 |
| Eaker, E. D., Pinsky, J. & Castelli, W. P. Myocardial infarction and coronary death among women: psychosocial predictors from a 20-year follow-up of women in the Framingham Study. Am. J. Epidemiol. 135, 854–864 (1992). | Age <65 only |
| Elovainio, M. et al. Contribution of risk factors to excess mortality in isolated and lonely individuals: an analysis of data from the UK Biobank cohort study. Lancet Public Health 2, e260–e266 (2017). | Age <50 |
| Eng, P. M., Rimm, E. B., Fitzmaurice, G. & Kawachi, I. Social ties and change in social ties in relation to subsequent total and cause-specific mortality and coronary heart disease incidence in men. Am. J. Epidemiol. 155(8):700-709 (2002) | Age <50 |
| Fleisch Marcus, A., Illescas, A. H., Hohl, B. C. & Llanos, A. A. Relationships between social isolation, neighborhood poverty, and cancer mortality in a population-based study of US adults. | Age <50 |
| Greenfield, T. K., Rehm, J. & Rogers, J. D. Effects of depression and social integration on the relationship between alcohol consumption and all-cause mortality. Addiction. 97, 29–38 (2002). | Age <50 |
| Gronewold, J. et al. Association of social relationships with incident cardiovascular events and all-cause mortality. Heart (Br. Card. Soc.) 106, 1317–1323 (2020). | Age <50 |
| Hakulinen, C. et al. Social isolation and loneliness as risk factors for myocardial infarction, stroke and mortality: UK Biobank cohort study of 479,054 men and women. Heart (Br. Card. Soc.) 104, 1536–1542 (2018). | Age <50 |
| Heffner, K. L., Waring, M. E., Roberts, M. B., Eaton, C. B. & Gramling, R. Social isolation, C-reactive protein, and coronary heart disease mortality among community-dwelling adults. Soc. Sci. Med. (1982) 72, 1482–1488 (2011). | No prospective cohort |
| Henriksen, J., Larsen, E. R., Mattisson, C. & Andersson, N. W. Loneliness, health and mortality. Epidemiol. Psychiatr. Sci. 28, 234–239 (2019). | Age <50 |
| Herlitz J, Wiklund I, Caidahl K, Hartford M, Haglid M, Karlsson B, et al. The feeling of loneliness prior to coronary artery bypass grafting might be a predictor of short-and long-term postoperative mortality. European Journal of Vascular and Endovascular Surgery. 1998;16(2):120–5. | Age <50 |
| Iecovich E, Jacobs JM, Stessman J. Loneliness, social networks, and mortality: 18 years of follow-up. Int J Aging Hum Dev. 2011;72(3):243-263. doi:10.2190/AG.72.3.e | Data not available for computing risk estimates |
| Iwasa H, Kawaai C, Gondo Y, Inagaki H, Suzuki T. [Subjective well-being and all-cause mortality among middle-aged and elderly people living in an urban Japanese community] Nihon Ronen Igakkai Zasshi. 2005;42:677–683. doi: 10.3143/geriatrics.42.67 | Not in English |
| Kammar-García A, Ramírez-Aldana R, Roa-Rojas P, et al. Association of loneliness and social isolation with all-cause mortality among older Mexican adults in the Mexican health and aging study: a retrospective observational study. BMC Geriatr. 2023;23(1):45. Published 2023 Jan 25. doi:10.1186/s12877-023-03750-3 | No prospective cohort |
| Kammar-García A, Ramírez-Aldana R, Roa-Rojas P, et al. Association of loneliness and social isolation with all-cause mortality among older Mexican adults in the Mexican health and aging study: a retrospective observational study. BMC Geriatr. 2023;23(1):45. Published 2023 Jan 25. doi:10.1186/s12877-023-03750-3 | No prospective cohort |
| Kandler U, Meisinger C, Baumert J, Löwel H, KORA Study Group Living alone is a risk factor for mortality in men but not women from the general population: a prospective cohort study. BMC Public Health. 2007;7:335. doi: 10.1186/1471-2458-7-335. | Age <50 |
| Kaplan, G. A. et al. Social connections and mortality from all causes and from cardiovascular disease: prospective evidence from eastern Finland. Am. J. Epidemiol. 128, 370–380 (1988). | Age <50 |
| Kim E, Sung K, Kim CO, Youm Y, Kim HC. The Effect of Cognitive Impairment on the Association Between Social Network Properties and Mortality Among Older Korean Adults. J Prev Med Public Health. 2023 Jan;56(1):31-40. doi: 10.3961/jpmph.22.350. Epub 2022 Nov 22. PMID: 36746420; PMCID: PMC9925289. | No prospective cohort |
| Kim YO, Lee W, Kim H, Cho Y. Social isolation and vulnerability to heatwave-related mortality in the urban elderly population: A time-series multi-community study in Korea. Environ Int. 2020;142:105868. doi:10.1016/j.envint.2020.105868 | Data not available for computing risk estimates |
| Koh-Bell, A., Chan, J., Mann, A. K. & Kapp, D. S. Social isolation, inflammation, and cancer mortality from the National Health and Nutrition Examination Survey—a study of 3,360 women. BMC Public Health | Age <50 |
| Kraav, S. L. et al. The effects of loneliness and social isolation on all-cause, injury, cancer, and CVD mortality in a cohort of middle-aged Finnish men: a prospective study. Aging Ment. Health 25, 2219–2228 (2021). | Age <50 |
| Kraav, S. L., Lehto, S. M., Kauhanen, J., Hantunen, S. & Tolmunen, T. Loneliness and social isolation increase cancer incidence in a cohort of Finnish middle-aged men: a longitudinal study. Psychiatry Res. 299, 113868 (2021). | Age <50 |
| Kroenke, C. H. et al. Social networks, social support, and burden in relationships, and mortality after breast cancer diagnosis in the Life After Breast Cancer Epidemiology (LACE) study. Breast Cancer Res. Treat. 137, 261–271 (2013). | Age <50 |
| Kroenke, C. H., Kubzansky, L. D., Schernhammer, E. S., Holmes, M. D. & Kawachi, I. Social networks, social support, and survival after breast cancer diagnosis. J. Clin. Oncol. 24, 1105–1111 (2006). | Age <50 |
| Laugesen K, Baggesen LM, Schmidt SAJ, et al. Social isolation and all-cause mortality: a population-based cohort study in Denmark. Sci Rep. 2018;8(1):4731. Published 2018 Mar 16. doi:10.1038/s41598-018-22963-w | Age <50 |
| Liu, J, and C Guo. *Beijing da xue xue bao. Yi xue ban = Journal of Peking University. Health sciences* vol. 54,2 (2022): 255-260. doi:10.19723/j.issn.1671-167X.2022.02.009 | Not in English |
| Luo Y, Waite LJ. Loneliness and mortality among older adults in China. J Gerontol B Psychol Sci Soc Sci. 2014;69(4):633-645. doi:10.1093/geronb/gbu007 | Data not available for computing risk estimates |
| Luo, J. & Hendryx, M. Mediation analysis of social isolation and mortality by health behaviors. Prev. Med 154, 106881 (2022). | Age <50 |
| Meller, Ingeborg, Manfred M. Fichter, and Hildegard Schröppel. "Riesgo de mortalidad en octogenarios y nonagenarios en relación con síntomas psíquicos, causa y lugar de fallecimiento: Resultados longitudinales de un estudio epidemiológico de seguimiento en una muestra comunitaria." *The European journal of psychiatry (edición en español)* 18.1 (2004): 45-61. | Not in English |
| Mollica RF, Sarajlic N, Chernoff M, Lavelle J, Vukovic IS, Massagli MP. Longitudinal study of psychiatric symptoms, disability, mortality, and emigration among Bosnian refugees. JAMA. 2001;286:546–554. doi: 10.1001/jama.286.5.546 | Age <50 |
| Naito, R. et al. Impact of social isolation on mortality and morbidity in 20 high-income, middle-income and low-income countries in five continents. BMJ Glob. Health | Age <50 |
| Obisesan, Thomas O. “Among elderly men, feelings of loneliness are associated with increased 10-year mortality risk, independent of social isolation and medical and psychiatric conditions.” *Evidence-based nursing* vol. 16,2 (2013): 66-7. doi:10.1136/eb-2012-100892 | No prospective cohort |
| Pantell, M. et al. Social isolation: a predictor of mortality comparable to traditional clinical risk factors. Am. J. Public Health 103, 2056–2062 (2013). | Age <50 |
| Pengpid S, Peltzer K, Anantanasuwong D. Longitudinal associations of loneliness with mental ill-health, physical ill-health, lifestyle factors and mortality in ageing adults in Thailand. BMC Psychiatry. 2023 Nov 17;23(1):855. doi: 10.1186/s12888-023-05263-0. PMID: 37978470; PMCID: PMC1065682 | Age <50 |
| Pinheiro, L. C., Reshetnyak, E., Akinyemiju, T., Phillips, E. & Safford, M. M. Social determinants of health and cancer mortality in the Reasons for Geographic and Racial Differences in Stroke (REGARDS) cohort study. Cancer 128, 122–130 (2022). | Age <50 |
| Rutledge, T. et al. Psychosocial predictors of long-term mortality among women with suspected myocardial ischemia: the NHLBI-sponsored Women’s Ischemia Syndrome Evaluation. J. Behav. Med. 39, 687–693 (2016 | Age <50 |
| Sarma, E. A. et al. Social integration and survival after diagnosis of colorectal cancer. Cancer 124, 833–840 (2018). | Age <50 |
| Schoenbach, V. J., Kaplan, B. H., Fredman, L. & Kleinbaum, D. G. Social ties and mortality in Evans County, Georgia. Am. J. Epidemiol. 123, 577–591 (1986). | Age <50 |
| Stringhini, S. et al. Socioeconomic status, structural and functional measures of social support, and mortality: the British Whitehall II Cohort Study, 1985–2009. Am. J. Epidemiol. 175, 1275–1283 (2012). | Age <50 |
| Takeuchi M, Showa S, Kitazawa K, Mori M. Living alone is associated with an increased risk of institutionalization in older men: a follow-up study in Hamanaka Town of Hokkaido, Japan. Geriatr Gerontol Int. 2018;18:867–872. doi: 10.1111/ggi.13267. | No outcome of interest |
| Tanskanen, J. & Anttila, T. A prospective study of social isolation, loneliness, and mortality in Finland. Am. J. Public Health 106, 2042–2048 (2016). | Age <50 |
| Terracciano A, Luchetti M, Karakose S, Stephan Y, Sutin AR. Loneliness and Risk of Parkinson Disease. JAMA Neurol. 2023 Nov 1;80(11):1138-1144. doi: 10.1001/jamaneurol.2023.3382. PMID: 37782489; PMCID: PMC10546293. | No outcome of interest |
| Udell JA, Steg PG, Scirica BM,  et al.  Living alone and cardiovascular risk in outpatients at risk of or with atherothrombosis.  Arch Intern Med. 2012;172(14):1086-1095 | Age <50 |

| Table S2. Characteristics of included studies for the association between loneliness and mortality | | | | | | | | | |
| --- | --- | --- | --- | --- | --- | --- | --- | --- | --- |
| **First Author** | **Year** | **Country** | **Sample Size** | **Age* (years)** | **Follow-up†** | **Exposure assessment** | **Outcome** | **Effect Size (95% CI)** | **Adjustment for covariates** |
| Barnes, et al. | 2022 | USA | Male 3533 Female 4449 | > 65 | 3 y | The revised UCLA Loneliness Scale (3-items) | All-cause mortality | HR 1.57 (1.28 to 2.93) | age, sex, education attainment, working status, income medication adherence, anxiety, CAD severity, |
| Blakoe, et al. | 2022 | Denmark | 7169 | 65.4 | 1 y | two questions in the DenHeart survey | All-cause mortality | HR 2.24 (1.24 to 4.03)/ HR 2.65 (1.32 to 5.32) | Age, educational level, comorbidity (Tu comorbidity index), smoking, alcohol intake, medicine compliance, body |
| Brandts, et al. | 2021 | Netherlands | Male 1032 Female 1078 | > 65 | 26 y | The De Jong Gierveld Loneliness Scale (11-items) | All-cause mortality | Male RR 0.99 (0.78 to 1.26) Female RR 1.10 (0.94 to 1.29) EL Male RR 0.95 (0.73 to 1.24) EL Female RR 1.01 (0.86 to 1.19) SL Male RR 1.01 (0.94 to 1.09) SL Female RR 0.91 (0.75 to 1.11) | age, educational level, marital status, number of (selected) diseases at baseline, MMSE-score, smoking status, body mass index, total physical activity per day, and number of alcoholic beverages per week |
| Chan, et al. | 2015 | Singapore | 4522 | ≥ 60 | 6d-4y | The three-item UCLA Loneliness Scale | All-cause mortality | HR 1.34 (0.99 to 1.82) | age, sex, education, living areas, ethnicity, smoking, drinking, activities of daily living, depressive symptoms, difficulties in IADL,cognitive function, comorbidity, social network, marital status, and housing type |
| Christensen, et al. | 2020 | Denmark | 13443 | >65 | 1 y | question about feeling lonely from the Danish National Health Survey | All-cause mortality | Male HR 2.14 (1.43 to 3.22) Female HR 2.92 (1.55 to 5.49) | age, living alone (when loneliness is the independent variable), loneliness (when living alone is the independent variable), cardiac diagnosis, educational level, comorbidity (Tu comorbidity index), BMI, smoking, alcohol intake and medicine compliance |
| Conde - Salla, et al. | 2020 | Europe | 48691 | ≥ 50 | 2 y | A short scale of Loneliness (3 items) | All-cause mortality | HR 1.19 (1.04 to 1.37) | age, sex, smoking, exercise self-rated health, activities of daily living, depressive symptoms, cognitive function comorbidity, marital status, frailty, and income |
| Crowe, et al. | 2021 | USA | 11302 | 50 - 95 | 8 y | The revised UCLA Loneliness Scale (3-items) | All-cause mortality | HR 1.48 (1.28 to 1.72) | age, sex, ethnicity, age-squared, and age-sex interactions |
| Cuijpers, | 2001 | Netherlands | 424 | 84.5 | 1y | De Jong Gierveld Loneliness Scale (12-items) | All-cause mortality | OR 1.03 (0.92 to 1.16) | gender, age, the number of years living in the residential home |
| Drageset, et al. | 2013 | Norway | Male 63 Female 164 | ≥ 65 | 5 y | The revised Social Provisions Scale (SPS), Emotional Loneliness | All-cause mortality | EL HR 0.96 (0.90 to 1.06) | sex, age group, educational level, marital status, length of stay, comorbidity, attachment, social integration, reassurance of worth, nurturance and GDS score |
| Elwardt, et al. | 2015 | Netherlands | Male 1498 Female 1413 | 54 - 85 | 20 y | 11-item De Jong Gierveld Loneliness Scale | All-cause mortality | EL HR 1.02 (0.99 to 1.06) SL HR 1.03 (0.99 to 1.07) | age, sex, depressive symptoms, cognitive function, physical health, physical activity, and anxiety |
| Gao, et al. | 2021 |  |  | ≥ 65 | 4 y | The Geriatric Mental State (GMS) - Automated Geriatric Examination for Computer Assisted Taxonomy (AGECAT) package: "Do you feel lonely?" (1-item) | All-cause mortality | Latin American countries HR 1.13 (1.01 to 1.26) | age, sex, education, depressive symptoms, household assets, social network, dependence, dementia, and living alone |
|  |  | Cuba | Cuba 2590 |  |  |  |  | Cuba HR 1.19 (0.97 to 1.46) |  |
|  |  | Dominican Republic | Dominican Republic 1696 |  |  |  |  | Dominican Republic HR 1.13 (0.92 to 1.39) |  |
|  |  | Peru | Peru 1707 |  |  |  |  | Peru HR 0.74 (0.49 to 1.11) |  |
|  |  | Venezuela | Venezuela 1679 |  |  |  |  | Venezuela HR 1.37 (0.96 to 1.94) |  |
|  |  | Mexico | Mexico 1833 |  |  |  |  | Mexico HR 1.11 (0.82 to 1.49) |  |
|  |  | Pureto Rico | Puerto Rico 1492 |  |  |  |  | Puerto Rico HR 1.10 (0.83 to 1.46) |  |
|  |  | China | China 1931 |  |  |  |  | China HR 1.58 (1.03 to 2.41) |  |
|  |  | India | India 745 |  |  |  |  | India HR 1.03 (0.65 to 1.62) |  |
| Gilmour, et al. | 2020 | Canada | 13037 | ≥ 65 | 8 - 9 y | The revised UCLA Loneliness Scale (3-items) | All-cause mortality | Male HR 0.9 (0.8 to 1.2) Female HR 1.1 (0.9 to 1.3) | age, education, living areas, smoking, physical health, physical activity, living alone, and marital status |
| Grand, et al. | 1990 | France | Male 290 Female 355 | ≥ 60 | 4 y | “Do you often feel lonely?” (1-item) | All-cause mortality | HR 1.42 (0.81 to 2.50) | age |
| Holwerda, et al. | 2016 | Netherlands | Male 1397 Female 1481 | 70.35 | 19 y | The De Jong Gierveld Loneliness Scale (11-items) | All-cause mortality | Male HR 1.13 (0.97 to 1.32) Female HR 0.99 (0.85 to 1.16) | age, sex, education, cardiovascular disease, depressive symptoms, cognitive function, chronic condition, social network, and marital status |
| Hong, et al. | 2023 | USA | 13752 | 67 | 4y | 11-item revised UCLA Loneliness Scale | All-cause mortality | HR 1.43 (1.17 to 1.76) | sociodemographic characteristics (age, sex, race/ethnicity, annual household income, total wealth, level of education, employment status, health insurance, geographic region), pre-baseline childhood abuse, pre-baseline values of the outcome variables (diabetes, hypertension, stroke, cancer, heart disease, lung disease, arthritis, overweight/obesity, physical functioning limitations, cognitive impairment, chronic pain, self-rated health, heavy drinking, current smoking status, physical activity, sleep problems, positive affect, optimism, purpose in life, mastery, health mastery, financial mastery, depressive symptoms, hopelessness, negative affect, perceived constraints), and personality factors (openness, conscientiousness, extraversion, agreeableness, neuroticism) |
| Hoogendijk, et al. | 2020 | Netherlands | Male 692 Female 735 | 75.7 | 22 y | The De Jong Gierveld Loneliness Scale (11-items) | All-cause mortality | HR 1.06 (0.92 to 1.22) | age, sex, education, smoking, depressive symptoms, chronic condition, and marital status |
| Julsing, et al. | 2016 | Netherlands | Male 719 | 64-85 | 25 y | The De Jong Gierveld Loneliness Scale (11-items) | All-cause mortality | HR 1.40 (0.85 to 2.31) | age, education, smoking, drinking, cardiovascular disease, chronic condition, physical activity, blood pressure, cholesterol, body mass index , dispositional optimism, and family history of stroke or myocardial infarction |
|  |  |  |  |  |  |  | CVD mortality | HR 1.18 (0.58 to 2.39) |  |
| Jylhä, et al. | 1989 | Finland | Male 472 Female 464 | 60-89 | 6.5 y | “Do you feel lonely?” (1-item) | All-cause mortality | Male HR 1.02 (0.75 to 1.40) Female HR 1.17 (0.79 to 1.74) | age, self-rated health, disability, and functioning |
| Lara, et al. | 2020 | Spain | 4467 | 47.7 | 6 y | The three-item UCLA Loneliness Scale | All-cause mortality | HR 1.02 (0.94 to 1.12) | age, sex, education, smoking, depressive symptoms, physical activity, body mass index , living alone, disability, and social participation |
| Lennartsson et al. | 2021 | Sweden | Male 475 Female 686 | ≥ 65 | 5 y | Are you ever bothered by feelings of loneliness? (1- item) | All-cause mortality | HR 1.20 (0.74 to 1.95) Male HR 1.14 (0.50 to 2.59) Female HR 1.11 (0.59 to 2.59) | age, sex, education, self-rated health, cardiovascular disease, social isolation, mobility, and psychological distress |
| Liang et al. | 2023 | China | 19360 | 59.4 (7.0) | 12.4y | The two-item UCLA Loneliness Scale | All-cause mortality/ CVD mortality | HR 1.00 (0.87 to 1.14)/ HR 1.03 (0.85 to 1.24) | demographic behavioural, psychological, physiological and diabetes-related factors, and comorbidities |
| Luo, et al. | 2012 | USA | Male 848 Female 1253 | ≥ 50 | 6 y | The revised UCLA Loneliness Scale (3-items) | All-cause mortality | HR 1.07 (0.99 to 1.17) | age, sex, education, ethnicity, smoking, exercise, self-rated health, depressive symptoms, household income, household assets, marital status, functioning, relatives living nearby, friends living nearby, and poor sleep quality |
| Maier, et al. | 1999 | Germany | Male 257 Female 256 | 70 - 103 | 5.17 y | The UCLA Loneliness Scale | All-cause mortality | RR 1.28 (1.14 to 1.44) | None |
| Moreno-Tamayo, et al. | 2022 | Mexico | 6393 | 64.5 | 12 y | Feeling of loneliness in the last 7 days (1-item) | All-cause mortality | HR 1.17 (1.05 to 1.29) | age, sex, education, living areas, smoking, exercise, activities of daily living (ADL), age × time, sex × time, health insurance, health insurance × time, depressive symptoms, difficulties in ADL × time, and difficulties in IADL |
| Newall, et al. | 2013 | Canada | 228 | 77 - 96 | 35 y | De Jong Gierveld Loneliness Scale (11-items) | All-cause mortality | HR 1.21 (1.07 to 1.36) | sex, age, marital status, health status, happiness, and income satisfaction |
| Ng, et al. | 2021 | Singapore | Male 2101 Female 2435 | > 60 | 6 y | UCLA loneliness scale (3-item) | All-cause mortality | Male HR 1.25 (0.92 to 1.7) Female HR 1.4 (1.05 to 1.86) | age, sex, ethnicity,  housing types, working  statuses, education,  perceived financial  adequacy, the number of  morbidities, ADL, IADL,  cognitive ability,  abdominal obesity, health  behaviors, social network  size, social engagement, and living arrangements |
| Novak, et al. | 2020 | Sweden | Male 240 Female 272 | 70 | 12 y | "Do you feel lonely?" (1-item) | All-cause mortality | HR 1.12 (1.01 to 1.24) Male HR 1.32 (0.77 to 2.28) Female HR 1.64 (0.98 to 2.76) | None |
|  |  |  |  |  |  |  | CVD mortality | Male HR 1.52 (0.78 to 2.96) Female HR 2.25 (1.14 to 4.45) |  |
| Novak, et al. | 2023 | Sweden | 778 | 70 | 6 y | "Do you feel lonely?" (1-item) | All-cause mortality | Male HR 2.24 (1.18 to 4.27) Female HR 0.63 (0.27 to 1.49) | perceived poor economic situation, hypercholesterolemia,impaired mobility,depression,current smoker,chronic bronchitis |
| Olaya, et al. | 2017 | Spain | Male 931 Female 1182 | 71.75 | 3 y | UCLA loneliness scale (3-item) | All-cause mortality | HR 1.00 (0.61 to 1.64) | age, sex, education, smoking, drinking, depressive symptoms, marital status, positive and negative support, and memory function |
| Olsen, et al. | 1991 | Denmark | Male 715 Female 1037 | 70-100 | 15 y | “Do you feel lonely?” (1-item) | CVD mortality | Male HR 1.70 (1.03 to 2.81) Female HR 1.09 (0.79 to 1.49) | age, self-rated health, mobility, physical activity, and psychiatric disease |
| OʼSúilleabháin, et al. | 2019 | Germany | Male 223 Female 190 | 84.53 (8.61) | 19 y | The Revised UCLA Loneliness Scale (8-items) | All-cause mortality | HR 1.18 (1.07 to 1.29) | None |
| Penninx, et al. | 1997 | Netherlands | 2829 | 55-84 | 3y | 11-item De Jong Gierveld Loneliness Scale | All-cause mortality | OR 1.06 (1.00 to 1.12) | age, sex, physical limitations, self-rated health, alcohol use, and smoking |
| Perissinotto, et al. | 2012 | USA | Male 651 Female 953 | 70.9 | 6 y | The Revised UCLA Loneliness Scale (3-items) | All-cause mortality | HR 1.45 (1.11 to 1.88) | age, sex, education, living areas, ethnicity, smoking, drinking, activities of daily living, depressive symptoms, comorbidity, physical activity, body mass index, income, net worth, working status, and hearing and vision problems |
| Pitkala, et al. | 2004 | Finland | Male 137 Female 354 | 75, 80, 85 | 10 y | “Do you suffer from loneliness?" (1-item) | All-cause mortality | HR 1.16 (0.99 to 1.39) | age, sex, and comprehensive health measure |
| Schutter, et al. | 2021 | Netherlands | Male 281 Female 162 | 60-93 | 6 y | The De Jong Gierveld Loneliness Scale (11-items) | All-cause mortality | HR 1.06 (0.99 to 1.14) | None |
| Shahtahmasebi, et al. | 1992 | UK | 534 | ≥65 | 8 y | Self-assessed loneliness (1-item) and loneliness measure (8- items) | All-cause mortality | HR 1.40 (0.99 to 1.99) | age |
| Shiovitz-Ezra, et al. | 2010 | USA | Male 3152 Female 4486 | 59.23 | 4 y | The item asks respondents whether they felt lonely much of the time over the past week | All-cause mortality | HR 1.83 (1.71 to 1.87) | age, sex, education, depressive symptoms, functioning, and medical status |
| Stek, et al. | 2005 | Germany | Male 171 Female 305 | 85 | 5 y | The De Jong Gierveld Loneliness Scale (11-items) | All-cause mortality | RR 1.3 (0.8 to 1.9) | age, sex, education, smoking, drinking, depressive symptoms, chronic condition, marital status, and institutionalized |
| Steptoe, et al. | 2013 | UK | Male 2953 Female 3547 | ≥52 | 7.25 y | The Revised UCLA Loneliness Scale (3-items) | All-cause mortality | HR 0.92 (0.78 to 1.09) | age, sex, education, ethnicity, diabetes, depressive symptoms, mobility, marital status, and wealth |
| Stessman, et al. | 2014 | Israel | Male 837 Female 729 | 70-90 | 20 y | How often do you feel lonely? (1-item) | All-cause mortality | HR 70–78: 1.06 (0.54 to 2.1) | sex, education, hypertension, diabetes, cardiovascular disease, and chronic condition |
| Stringhini, et al. | 2018 | UK | Male 4291 Female 3555 | 66.4 (9.6) | 8.4 y | An abridged version of the 20-item Revised UCLA Loneliness Scale | All-cause mortality | HR 1.23 (1.10 to 1.38) | age, sex, smoking, drinking, hypertension, physical activity, hs-CRP, cholesterol, body mass index (BMI), social network, triglycerides level, fibrinogen level, and positive and negative support |
|  |  |  |  |  |  |  | CVD mortality | HR 1.44 (1.29 to 1.74) |  |
| Tabue Teguo et al. | 2016 | France | Male 1524 Female 2096 | 76.5 (7.1) | 22 y | Item 14 (I felt lonely) of the French version of the Centre for Epidemiological Studies Depression Scale "Do you suffer from loneliness?" | All-cause mortality | HR 1.11 (1.00 to 1.23) | age, sex, education, diabetes, cardiovascular disease, angina pectoris, and respiratory problems |
| Tilvis, et al. (a) | 2012 | Finland | Male 1187 Female 2671 | > 75 | 7 y | “Do you suffer from loneliness?" (1-item) | All-cause mortality | Male HR 1.39 (1.15 to 1.68) Female HR 1.16 (1.01 to 1.33) | age, social isolation, living alone, and social inactivity |
| Tilvis, et al. (b) | 2012 | Finland | Male 812 Female 1678 | ≥ 75 | 4.75y | “Do you suffer from loneliness?" (1-item) | All-cause mortality | HR 1.18 (0.99 to 1.42) | age, sex, depressive symptoms, satisfied with life, feeling needed, plans for future, and zest for life |
| Tilvis, et al. | 2011 | Finland | Male 1131 Female 2556 | 81 | 4.75y | “Do you suffer from loneliness?" (1-item) | All-cause mortality | HR 1.17 (1.02 to 1.33) | age, sex, and self-rated health |
| Wang, et al. | 2020 | UK | Male 207 Female 458 | 81-103 | 10 y | "Do you feel lonely?" (1-item) | All-cause mortality | HR 1.00 (0.80 to 1.40) | age, sex, education, living areas, depressive symptoms, chronic condition, physical health, and marital status |
| Ward, et al. | 2021 | Ireland | 6915 | ≥ 50 | 7 y | A modified version of the University of California Los Angeles Loneliness scale | All-cause mortality | HR 1.26 (0.94 to 1.68) | sex, education, living areas, smoking, drinking, cardiovascular disease, depressive symptoms, chronic condition, walk minutes, obese, and polypharmacy |
|  |  |  |  |  |  |  | Cancer mortality | HR 1.02 (0.68 to 1.53) |  |
|  |  |  |  |  |  |  | Circulatory system mortality | HR 1.28 (0.77 to 2.12) |  |
| Wei, et al. | 2022 | China | 10993 | ≥65 | 3y | Do you feel lonely? (1- item) | All-cause mortality | HR 1.10 (1.02 to 1.19) | age, gender, race, marital status, residence, occupation, education, BMI, smoking, alcohol drinking, living preference, socioeconomic status, dietary habits, social/leisure activity score, physical exercise, poor self-rated health, poor interviewer-rated health, comorbidities (≥2), hypertension, diabetes, heart disease, stroke, serious illness in the past 2 years, hearing problem, visual impairment, cognitive impairment, functional limitation, and frailty |
| Youm, et al. | 2021 | Korea | Male 289 Female 390 | 73.9 (7.5) | 5.61 y | The Center for Epidemiologic Studies Depression Scale (CES-D) (1-item) | All-cause mortality | HR 1.01（0.43 to 2.36） | sex, education, smoking, drinking, psychological well-being, cognitive function, household income, social disengagement, segregation, living with spouse, physical health, and comorbidity |
| Yu , et al. | 2020 | China | Male 645 Female 622 | ≥65 | 10 y | The Chinese version Center for Epidemiologic Studies Depression Scale (CES-D) | All-cause mortality | HR 0.95 (0.82 to 1.09) | age, educational level , comorbidity, smoking , alcohol intake,body mass index , sex , working status , income , physical activity , activities of daily living (ADL), and depressive symptoms |
| Yu, et al. | 2022 | China | Male 19768 Female 15486 | 86.63 (11.39) | 4.8 y | How often do you feel lonely? (1-item) | All-cause mortality | HR 1.01 (0.98 to 1.04) Male HR 1.01 (0.97 to 1.05) Female HR 1.01 (0.96 to 1.06) | age, sex, education, living areas, ethnicity, smoking, drinking, exercise, self-rated health, activities of daily living, psychological well-being, hypertension, diabetes, cardiovascular disease, and social isolation |
| Yu X, et al. | 2023 | USA | 9032 (5646 F) | 63.99 (8.62) | 8y | Do you feel lonely? (yes vs. no), which is an item from the 8-item CES-D Scale | All-cause mortality | HR 1.05 (0.96 to1.15) HR1.06 (0.95 to 1.19) HR 1.16 (1.02 to 1.33) | gender, race/ethnicity, marital status, education, employment status, household wealth, objective social isolation index, obesity, CES-D scores, activities of daily living scores, self-rated health, and the number of comorbid diseases |
| Zhen, et al. | 2015 | China | 3089 | ≥ 65 | 3 y | Sense of loneliness (1- item) | All-cause mortality | HR 1.18 (1.1 to 1.27) | sex, age, education, marital status, smoking, race/ethnicity, urban/ rural, income, and covered by public medical service |

| Table S3. Characteristics of included studies for the association between social isolation and mortality | | | | | | | | | |
| --- | --- | --- | --- | --- | --- | --- | --- | --- | --- |
| **First Author** | **Year** | **Country** | **Sample Size** | **Age* (years)** | **Follow-up†** | **Exposure assessment** | **Outcome** | **Effect Size HR/RR (95% CI)** | **Adjustment for covariates** |
| Barnes, et al. | 2022 | USA | Male 3533 Female 4449 | >65 | 3y | Social network index | All-cause mortality | HR 2.47 (1.91 to 3.18) | age, sex, living areas, psychological distress, health checkup, nurturance, and functioning |
| Chan, et al. | 2015 | Singapore | 4522 | ≥60 | 1,459 d | Social network scale | All-cause mortality | HR 1.28 (0.90 to 1.82) | age, sex, living areas, ethnicity, smoking, social isolation, health insurance, chronic condition, hierarchical condition category (HCC) score, and fibrinogen level |
| Crowe, et al. | 2021 | USA | 11302 | 50-95 | 8 y | A 6-item social isolation scale | All-cause mortality | HR 1.38 (1.20 to 1.57) | age, sex, ethnicity, age-squared, and age-sex interactions |
| Drageset, et al. | 2013 | Norway | Male 63 Female 164 | ≥65 | 5 y | Social provisions scale | All-cause mortality | HR 1.02 (0.96 to 1.08) | age, sex, living areas, ethnicity, social isolation, blood pressure, cholesterol, body mass index (BMI), household assets, and social network |
| Gilmour, et al. | 2020 | Canada | Male 5408 Female 7629 | ≥65 | 8-9 y | Eight community-related activities | All-cause mortality | Male HR 1.30 (1.10 to 1.50) Female HR 1.40 (1.20 to 1.60) | age, living areas, ethnicity, health insurance, health checkup, psychological distress, and positive affect |
| Elwardt, et al. | 2015 | Germany | 2911 | 55 – 85 | 17 y | Cohen’s Social Network Index | All-cause mortality | HR 0.95 (0.92 to 0.98) | age + single predictor + mental health + cognitive health + physical health |
| Holwerda, et al. | 2012 | Netherlands | Male 1509 Female 2495 | 65-84 | 10 y | Marital status, living arrangement and social network | All-cause mortality | Male RR 1.37 (1.19 to 1.59) Female RR 1.29 (1.14 to 1.45) | living alone |
| Hong, et al. | 2023 | USA | 13752 | 67 | 4y | 8-item social isolation measure | All-cause mortality | HR 1.76 (1.25 to 2.42) | sociodemographic characteristics (age, sex, race/ethnicity, annual household income, total wealth, level of education, employment status, health insurance, geographic region), pre-baseline childhood abuse, pre-baseline values of the outcome variables (diabetes, hypertension, stroke, cancer, heart disease, lung disease, arthritis, overweight/obesity, physical functioning limitations, cognitive impairment, chronic pain, self-rated health, heavy drinking, current smoking status, physical activity, sleep problems, positive affect, optimism, purpose in life, mastery, health mastery, financial mastery, depressive symptoms, hopelessness, negative affect, perceived constraints), and personality factors (openness, conscientiousness, extraversion, agreeableness, neuroticism) |
| Hoogendijk, et al. | 2020 | Netherlands | Male 692 Female 735 | 75.7 | 22 y | partner status, social support and network | All-cause mortality | HR 1.06 (0.92 to 1.21) | age , sex , education, diagnosed depression, smoking status, and chronic disease |
| Jenkinson, et al. | 1993 | UK | Male 1073 Female 303 | X | 3y | social isolation scale | All-cause mortality | HR 1.33 (0.89 to 1.98) | age , sex ,diabetes history hypertension history,previous documented infarct, hospital complications, and car owner |
| Jeong, et al. | 2022 | Korea | Male 320 Female 713 | **>** 60 | 2 y | Abbreviated version of the Lubben Social Network Scale | All-cause mortality | Male RR 4.50 (1.90 to 9.90) Female RR 1.20 (0.05 to 2.80) | age, cohabitation status, education level, hypertension, diabetes mellitus, and depressive symptom scores at baseline |
| Jylhä, et al. | 1989 | Finland | Male 472 Female 464 | 60-89 | 6.5 y | index of social participation | All-cause mortality | Male HR 1.15 (0.84 to 1.57) Female HR 1.38 (0.88 to 2.16) | age, self-rated health, disability, and functioning |
| Keller, et al | 2003 | USA | Male 370 Female 900 | 50-97 | 10 y | social network index | All-cause mortality | HR 1.25 (0.97 to 1.61) | age, education, income level, basic activities of daily living (ADL), cumulative illness rating scale (CIRS), informal service use |
| Kornej, et al. | 2022 | USA | 3454 | ≥ 55 (67.4) | 11.8 y | Berkam-Syme SNI quesionnaire | All-cause mortality | HR 1.62 (1.14-2.29) | age, sex, height, weight, systolic blood pressure, diastolic blood pressure, hypertension treatment, current smoking, diabetes, history of myocardial infarction, and history of heart failure and competing risk of AF |
| Kotozaki, et al. | 2022 | Japan | Male 8059 Female 14874 | 58.8 | 5 y | LSNS-6 | All-cause mortality | Male HR 1.38 (1.05 to 1.83) Female HR 1.49 (1.02 to 2.19) | age, area, education level, working status, marital status, number of household members, smoking habits, drinking habits, exercise habits, BMI, medical history, depressive symptoms, AIS, SC, house damage due to the GEJE, and death of family members due to the GEJE |
| Kreibig, et al. | 2014 | USA | 1019 | 63.4 | 6.7 y | Social network index | All-cause mortality | HR 1.36 (0.91 to 2.04) | sex, education attainment, income, alcohol consumption, physical activity, body mass index, charlson comorbidity index, depressive symptoms, ethnicity, cardiac disease severity, risk factors, medication use, stress-related, inflammatory, glycemic, cholesteric biomarkers, diet, subjective sleep quality, medication adherence, anxiety |
| Kroenke, et al. | 2020 | USA | Female 1431 | 50-79 | 5.8 y | Social network index | All-cause mortality | HR 1.27 (0.97 to 1.66) | age, time between diagnosis and assessment of social networks, cancer stage at diagnosis, chemotherapy, radiation, use of hormone replacement therapy, smoking status, body mass index, physical activity, education, race, alcohol intake, surgery, basic activities of daily living (ADL), cancer grade, cancer site, study, income, family history of colorectal cancer, comorbidity, depressive symptoms, colorectal cancer screening |
|  |  |  |  |  |  |  | Colorectal cancer mortality | HR 1.14 (0.81 to 1.61) |  |
| Kroenke, et al. | 2017 | USA | Female LACE cohort: 1947 NHS cohort: 2221 SBCSS cohort: 2127 WHEL cohort: 2972 | 56.1 | 1.8 y | Social network index | All-cause mortality | HR (LACE NHS, SBCSS) 1.69 (1.43 to 1.99) HR (WHEL) 1.16 (0.90 to 1.51) | loneliness, body mass index, occupational class, education, race, HER-2 neu status, cancer grade, comorbidity, and cohort |
|  |  |  |  |  |  |  | Breast cancer mortality | HR (LACE NHS, SBCSS) 1.64 (1.33 to 2.03) HR (WHEL) 1.00 (0.75 to 1.34) |  |
| Liang et al. | 2023 | China | 19360 | 59.4 (7.0) | 12.4y | The social isolation index score | All-cause mortality | HR 1.33 (1.19 to 1.47) | demographic behavioural, psychological, physiological and diabetes-related factors, and comorbidities |
|  |  |  |  |  |  |  | CVD mortality | HR 1.36 ( 1.17 to 1.59) |  |
| Lennartsson et al. | 2021 | Sweden | Male 475 Female 686 | ≥ 65 | 5 y | Social isolation index | All-cause mortality | HR 2.54 (1.58 to 4.04) Male HR 2.77 (1.50 to 5.12) Female HR 2.73 (1.23 to 6.05) | age, sex, education, self-rated health, cardiovascular disease, social isolation, mobility, and psychological distress |
| Lund, et al. | 2000 | Denmark | 894 | NR | 4 y | Number of monthly contacts | All-cause mortality | OR 3.78 (1.08 to 13.20) | age (70 - 74 years), functional ability, self-rated health, and contact frequency + self-rated health*age |
|  |  |  |  |  |  |  | All-cause mortality | OR 0.69 (0.33 to 1.43) | age (75+ years), functional ability, self-rated health, and contact frequency + self-rated health*age |
| Manemann, et al | 2018 | USA | 1681 | 73.29 | 0.67y | Social isolation short form | All-cause mortality | HR 3.74 (1.82 to 7.70) | age, sex, education attainment, charlson index, marital status |
| Moreno-Tamayo, et al. | 2022 | Mexico | 6393 | 64.5 | 12 y | In house five items | All-cause mortality | HR 1.24 (1.10 to 1.38) | age, sex, education, living areas, smoking, exercise, activities of daily living (ADL), age × time, sex × time, health insurance, health insurance × time, depressive symptoms, difficulties in ADL × time, and difficulties in IADL |
| Murberg, et al. | 2004 | Norway | Male 85 Female 34 | 65.7-66.8 | 6y | In house four items | All-cause mortality | HR 1.36 (1.04 to 1.78) | age, sex, NYHA (is the most widely used measure of functional status in individuals with chronic heart disease), neuroticism, and charlson index |
| Rutledge, et al. | 2004 | USA | 503 | 59 | 2.3y | Social Network Index (SNI; 17) | All-cause mortality | RR 2.40 (1.04 to 5.40) | Age, depression, smoking status, atherosclerosis severity and income level |
| Saito, et al. | 2021 | Japan/UK | 15313 | ≥65 | 10 y | Frequency of contacts with family, friends or neighbors and Isolation risk score | All-cause mortality | JAGES HR 1.18 (1.05 to 1.33) ELSA 1.27 (0.85 to 1.89) JAGES HR 1.30 (1.12 to 1.50) ELSA 2.05 (1.52 to 2.73) | Sex, age, self‐rated health, presence of medical treatment, marital status, equivalent income and basic activities of daily living at baseline |
| Saito, et al. | 2012 | Japan | 12864 | ≥65 | 4 y | Both face-to face and non-face-to-face contacts | All-cause mortality | HR 1.19 (1.02 to 1.39) Male HR 1.02 (0.85 to 1.24) Female HR 1.72 (1.31 to 2.25) | educational attainment, marital status, disease and/or impairment, and municipality of residence |
| Sakurai, et al. | 2019 | Japan | 1023 | ≥65 | 6 y | Questionnaire (frequency of contacts with family, friends, neighbors | All-cause mortality | HR 1.34 (0.64 to 2.81) | age, sex, number of years of education, comorbidities, depression symptoms, subjective health, and residential areas |
| Seeman, et al. | 1993 | USA | 9635 | ≥65 | 5 y | Presence of spouse, contact with two or more close friends and/or relatives, church attendance and membership in groups | All-cause mortality | East Boston | age, no. of chronic conditions, BMI, angina, Katz disability |
|  |  |  |  |  |  |  |  | Male HR 0.99 (0.66 to 1.51) |  |
|  |  |  |  |  |  |  |  | Female HR 1.28 (0.79 to 2.03) |  |
|  |  |  |  |  |  |  |  | Iowa |  |
|  |  |  |  |  |  |  |  | Male HR 1.42 (0.87 to 2.32) |  |
|  |  |  |  |  |  |  |  | Female HR 1.89 (1.18 to 3.04) |  |
|  |  |  |  |  |  |  |  | New Havem |  |
|  |  |  |  |  |  |  |  | Male HR 2.40 (1.35 to 3.07) |  |
|  |  |  |  |  |  |  |  | Female HR 1.78 (1.05 to 3.03) |  |
| Smith et al. | 2018 | UK | 7731 | 64.0 (9.5) | 8y | index of social isolation | All-cause mortality | HR 1.29 (1.10 to 1.51) | ethnicity, educational attainment, occupational class, wealth, quintile long-standing illness, functional impairment, depressive symptoms, self-reported doctor-diagnosed disease: heart disease, diabetes, cancer, stroke, asthma, and chronic lung disease |
| Tilvis, et al. (a) | 2012 | Finland | Male 1187 Female 2671 | > 75 | 7 y | six in house questions | All-cause mortality | Male HR 1.03 (0.87 to 1.23) Female HR 1.00 (0.87 to 1.15) | age |
| Wang, et al. | 2022 | China | 30430 | > 60 | 13 y | Face-to-face contact with co-inhabitants | CVD mortality | HR 1.61 (1.20 to 2.03) | age, sex, self-rated health, socioeconomic position, biological factors, behavioral factors |
|  |  |  |  |  |  |  | Cancer mortality | HR 1.01 (0.71 to 1.33) |  |
|  |  |  |  |  |  |  | Other mortality | HR 1.30 (0.93 to 1.39) |  |
|  |  |  |  |  |  | Face-to-face contact with non-co-inhabitants | CVD mortality | HR 1.91 (1.20 to 2.62) |  |
|  |  |  |  |  |  |  | Cancer mortality | HR 1.36 (0.79 to 1.93) |  |
|  |  |  |  |  |  |  | Other mortality | HR 1.57 (0.83 to 2.11) |  |
|  |  |  |  |  |  | Non-face-to-face contact (by telephone/mail) | CVD mortality | HR 1.30 (1.08 to 1.56) |  |
|  |  |  |  |  |  |  | Cancer mortality | HR 1.14 (0.93 to 1.19) |  |
|  |  |  |  |  |  |  | Other mortality | HR 1.37 (1.12 to 1.67) |  |
|  |  |  |  |  |  | Club/organization contact | CVD mortality | HR 1.05 (0.94 to 1.16) |  |
|  |  |  |  |  |  |  | Cancer mortality | HR 0.90 (0.81 to 1.00) |  |
|  |  |  |  |  |  |  | Other mortality | HR 1.01 (0.90 to 1.13) |  |
| Ward, et al. | 2021 | Ireland | 6915 | ≥ 50 | 7 y | Social network index | All-cause mortality | HR 1.43 (1.09 to 1.87) Male HR 1.18 (0.79 to 1.75) Female HR 1.66 (1.08 to 2.54) | sex, education, living areas, smoking, drinking, cardiovascular disease, depressive symptoms, chronic condition, walk minutes, obese, and polypharmacy |
|  |  |  |  |  |  |  | Cancer mortality | HR 1.18 (0.82 to 1.69) |  |
|  |  |  |  |  |  |  | Circulatory system mortality | HR 1.19 (0.75 to 1.88) |  |
| Yang, et al. | 2013 | USA | Male 3082 | NR | 18 y | number of social ties across four domains: marriage, contacts with friends and relatives, religious attendance, and membership in social organizations | All-cause mortality | Male HR 1.53 (1.26 to 2.19) | age, race ethnicity, education, family income, smoking, drinking, physical activity, BMI, chronic conditions, self-rated health |
|  |  |  | Female 3647 |  |  |  |  | Female HR 1.45 (1.20 to 1.75) |  |
|  |  |  | Male 3082 |  |  |  | Circulatory system | Male HR 1.56 (1.16 to 2.10) |  |
|  |  |  | Female 3647 |  |  |  |  | Female HR 1.47 (1.11 to 1.95) |  |
|  |  |  | Male 3082 |  |  |  | Cancer | Male HR 1.30 (0.87 to 1.95) |  |
|  |  |  | Female 3647 |  |  |  |  | Female HR 0.86 (0.55 to 1.34) |  |
| Youm, et al. | 2021 | Korea | Male 289 Female 390 | 73.9 (7.5) | 5.61 y | Levels of participation | All-cause mortality | HR 1.53 (0.82 to 2.84) | sex, education, smoking, drinking, psychological well-being, cognitive function, household income, social disengagement, segregation, living with spouse, physical health, and comorbidity |
| Yu, et al. | 2022 | China | Male 19768 Female 15486 | 86.63 (11.39) | 4.8 y | In house five items | All-cause mortality | HR 1.22 (1.18 to 1.25) Male HR 1.19 (1.14 to 1.23) Female HR 1.27 (1.22 to 1.33) | age, sex, education, living areas, ethnicity, smoking, drinking, exercise, self-rated health, activities of daily living, psychological well-being, hypertension, diabetes, cardiovascular disease, and social isolation |
| Yu , et al. | 2020 | China | Male 645 Female 622 | ≥65 | 10 y | Social isolation index | All-cause mortality | HR 1.16 (1.06 to 1.26) | age , educational level , comorbidity, smoking , alcohol intake,body mass index , sex , working status , income , physical activity , activities of daily living (ADL), and depressive symptoms |

| Table S4. Characteristics of included studies for the association between living alone and mortality | | | | | | | | | |
| --- | --- | --- | --- | --- | --- | --- | --- | --- | --- |
| **First Author** | **Year** | **Country** | **Sample Size** | **Age* (years)** | **Follow-up†** | **Exposure assessment** | **Outcome** | **Effect Size HR/RR (95% CI)** | **Adjustment for covariates** |
| Abell, et al. | 2021 | UK | 4888 | 68.6 | 8.5 y | number of people recorded in each household | All-cause mortality | HR 1.20 (1.04 to 1.38) | age, sex, education and wealth, depressive symptoms, chronic disease/limiting long term illness, health behaviour, mobility impairment, loneliness |
| Avlund, et al. | 1998 | Denmark | Male 362 Female 365 | 70 | 11 y | Live alone, live with others | All-cause mortality | Male OR 2.11 (1.10 to 4.10) Female OR 1.43 (0.90 to 2.30) | No adjustment |
| Chan, et al. | 2015 | Singapore | 4522 | ≥60 | 4y | information from the household roster | All-cause mortality | HR 0.71 (0.35 to 1.41) | Individual-level, household-level and societal-level loneliness, socio-demographic values and health status indicators |
| Dahl, et al. | 2021 | Norway | Male 12770 | > 68 | 12.8 y | Number of people in each household | All-cause mortality | Male HR 1.59 (1.41 to 1.78) | age at Census, education level, urbanization degree and no. of children |
|  |  |  | Female 22067 |  |  |  |  | Female HR 1.22 (1.13 to 1.32) |  |
| Gopinath, et al. | 2013 | Australia | 3508 | > 49 | 10 y | Participants were asked who lived with them; if they responded that they lived with nobody or with pets only, they were classified as living alone | All-cause mortality | HR 1.18 (0.98 to 1.43) | Age, sex, education, current smoking, body mass index, walking disability, prior diagnosis of heart disease, angina, heart attack, diabetes mellitus,cancer, poor self-rated health, and 36-Item Short-Form Survey mental and physical component summary scores |
|  |  |  |  |  |  |  |  |  |  |
| Jensen, et al. | 2019 | Denmark | 3346 | 62.9 | 10 y | Self-report living alone or not | All-cause mortality | HR 1.23 (1.09 to 1.39) |  |
|  |  |  |  |  |  |  |  |  | age, previous cardiovascular disease (stroke or myocardial infarction), presence of diabetes, body mass index, systolicblood pressure, smoking, alcohol, self-reported physical activity, se-triglycerides, se-total cholesterol, resting heart rate, workers compensation, satisfaction with current housing situation, mood, self-reported health, and socioeconomic position. |
|  |  |  |  |  |  |  | Cardiovascular mortality | HR 1.36 (1.13 to 1.63) |  |
|  |  |  |  |  |  |  |  |  |  |
| Jiang, et al. | 2023 | USA | 3154 | 72.81 | 10y | Number of people in each household | All-cause mortality | HR 1.14 (0.95 to 1.38) | Age, sex, sociodemographic covariates, health and behavioral covariates, loneliness, social engagement and depression |
| Jylhä, et al. | 1989 | Finland | Male 472 Female 464 | 60-89 | 6.5 y | living alone: ('How many people are living here with you?'). | All-cause mortality | Male HR 0.98 (0.70 to 1.37) Female HR 0.85 (0.58 to 1.26) | age, self-rated health, disability, and functioning |
| Khalatbari – Soltani, et al. | 2020 | Australia | 1522 | ≥70 | 9 y | living arrangement variables were used: living alone (yes/no) and living with children or grandchildren (yes/no) | All-cause mortality | HR 1.50 (1.26 to 1.77) | age, age squared, and country of birth |
|  |  |  |  |  |  |  | CVD mortality | HR 1.41 ( 0.99 to 1.98) |  |
|  |  |  |  |  |  |  | Cancer mortality | HR 1.36 ( 0.98 to 1.89) |  |
|  |  |  |  |  |  |  | non-CVD, non-cancer mortalitY | HR 1.76 (1.29 to 2.39) |  |
| Koivunen, et al. | 2020 | Finland | 558 | NR | 3.8 y | Living alone versus living with someone | All-cause mortality | Male HR 1.27 (0.82 to 1.75) | age, loneliness and self-rated health at baseline |
|  |  |  |  |  |  |  |  | Female HR 0.84 (0.64 to 1.09) |  |
| Ng, et al. | 2015 | Singapore | Male 935 Female 1638 | 65.9 | 8y | Who do you live with? | All-cause mortality | HR 1.66 (1.05 to 2.63) Male HR 2.36 (1.24 to 4.49) Female HR 1.14 (0.58 to 2.22) | socio-demographic and health status and marital status |
| Novak, et al. | 2023 | Sweden | 778 | 70 | 6 y | Living alone was categorized as individuals who are single, or divorced, or widowed and live alone | All-cause mortality | Male HR 2.56 (1.27 to 5.16) Female HR 0.73 (0.28 to 1.90) | perceived poor economic situation, hypercholesterolemia,impaired mobility,depression,current smoker,chronic bronchitis |
| Pimouguet, et al. | 2016 | Sweden | 2404 | ≥66 | 6 y | Question “Are you living alone?” | All-cause mortality | HR 1.35 (1.18 to 1.54) | age, sex, education, recent financial difficulty, BMI, smoking |
|  |  |  |  |  |  |  |  |  | habits, alcohol consumption, diabetes, hypertension, stroke, heart failure, coronary heart disease, depression, dementia, cancer, ADL and |
|  |  |  |  |  |  |  |  |  | IADL disability, MMSE, feeling of loneliness and institutionalization |
| Renwick, et al. | 2020 | USA | 15788 | ≥55 | 10 y | CCHS with | All-cause mortality | ≥55 HR 1.08 (1.00 to 1.16) | Univariate, age, sex, income, smoking status, frailty |
|  |  |  |  |  |  | 10 possible household configurations that included living alone and some combination of a spouse, parents, |  | 55 – 64 HR 1.17 (0.99 to 1.39) |  |
|  |  |  |  |  |  | children, siblings, or other |  | ≥65 HR 1.06 (0.97 to 1.15) |  |
| Scafato, et al. | 2008 | Italy | 5376 | 65 – 84 | 10 y | Questions “Which is your marital status?” and “Are you living with others?” | All-cause mortality | Male HR 1.42 (1.05 to 1.92) | age, |
|  |  |  |  |  |  |  |  | Female HR 1.05 (0.81 to 1.35) | SBP, DBP, GLUC, CHOL, HDL-CH, BMI, education, procreation, smoking |
|  |  |  |  |  |  |  |  |  | habit, alcohol use, ADLs, IADLs, depression and cognitive impairment |
| Takeuchi, et al. | 2018 | Japan | 539 | 70 – 85 | 2 y | Living alone versus living with someone | All-cause mortality | HR 1.28 (0.52 to 3.13) | Age, sex, daily support from family, history of hypertension, cancer, cerebral apoplexy or pneumonia |
|  |  |  |  |  |  |  |  | Male HR 0.65 (0.11 to 3.74) |  |
|  |  |  |  |  |  |  |  | Female HR 2.18 (0.73 to 6.55) |  |
| Wei, et al. | 2022 | China | 10993 | ≥65 | 3y | One question “Who do you live with?” with responses including ‘living with family (including house maid)’ and ‘living alone (LA) | All-cause mortality | HR 1.07 (0.91 to 1.25) | age, gender, race, marital status, residence, occupation, education, BMI, smoking, alcohol drinking, living preference, socioeconomic status, dietary habits, social/leisure activity score, physical exercise, poor self-rated health, poor interviewer-rated health, comorbidities (≥2), hypertension, diabetes, heart disease, stroke, serious illness in the past 2 years, hearing problem, visual impairment, cognitive impairment, functional limitation, and frailty |

| Table S5. New-castle-Ottawa scores for the included studies | | | | | | | | | | |
| --- | --- | --- | --- | --- | --- | --- | --- | --- | --- | --- |
|  |  | **Selection** | | | | **Comparability** | **Outcome** | | |  |
|  | | | | | | | | | |  |
| Study | Year | Representativeness | Selection of the non-exposed cohort | Ascertainment of exposure | Outcome | Comparability | Assessment of outcome | Adequate follow up time | Adequacy of follow-up cohort | Score |
| Abell, et al. | 2021 | * | * | * | * | ** | * | * | * | 9 |
| Avlund, et al. | 1998 | * | * | * | * | - | * | * | * | 7 |
| Barnes, et al. | 2022 | - | * | * | * | * | * | * | - | 6 |
| Blakoe, et al. | 2022 | - | * | * | * | * | * | * | - | 6 |
| Brandts, et al. | 2021 | * | * | * | * | ** | * | * | * | 9 |
| Chan, et al. | 2015 | * | * | * | * | * | * | * | * | 8 |
| Christensen, et al. | 2020 | - | * | - | * | * | * | * | * | 6 |
| Conde-Sala, et al. | 2020 | * | * | * | * | * | * | - | * | 7 |
| Crowe, et al. | 2021 | * | * | * | * | * | * | * | * | 8 |
| Cuijpers, | 2001 | * | - | * | * | ** | * | * | * | 8 |
| Dahl, et al. | 2021 | * | * | * | * | ** | - | * | - | 7 |
| Drageset, et al. | 2013 | - | * | * | * | * | * | * | * | 7 |
| Elwardt, et al. | 2015 | * | * | * | * | * | * | * | * | 8 |
| Gao, et al. | 2021 | * | - | * | * | * | - | * | * | 6 |
| Glimour, et al. | 2020 | * | * | * | * | * | * | * | * | 8 |
| Gopinath, et al. | 2013 | * | * | - | * | ** | * | * | * | 8 |
| Grand, et al. | 1990 | - | * | - | * | * | * | * | * | 6 |
| Holwerda, et al. | 2012 | * | * | - | * | * | * | * | * | 7 |
| Holwerda, et al. | 2016 | * | * | * | * | * | * | * | * | 8 |
| Hong, et al. | 2023 | * | * | * | * | ** | - | - | * | 7 |
| Hoogendijk, et al. | 2020 | * | * | * | * | * | * | * | * | 8 |
| Jenkinson, et al. | 1993 | - | * | * | * | * | * | * | * | 7 |
| Jensen, et al. | 2019 | - | * | * | * | ** | - | * | * | 7 |
| Jeong et al. | 2022 | - | * | * | * | * | - | * | * | 6 |
| Jiang, et al. | 2023 | * | * | - | * | ** | - | * | - | 6 |
| Julsing, et al. | 2016 | * | * | * | * | * | * | * | * | 8 |
| Jylhä, et al. | 1989 | * | * | - | * | * | * | * | * | 7 |
| Keller, et al. | 2003 | - | * | * | * | * | * | * | * | 7 |
| Khalatbari – Soltani, et al. | 2020 | * | * | * | * | * | * | * | * | 8 |
| Koivunen, et al. | 2020 | * | * | - | * | ** | * | * | * | 8 |
| Kornej et al. | 2022 | - | * | * | * | * | * | * | * | 7 |
| Kotozaki, et al. | 2022 | - | * | * | * | * | - | * | * | 6 |
| Kreibig, et al. | 2014 | - | * | * | * | * | * | * | * | 7 |
| Kroenke, et al. | 2017 | - | * | * | * | * | * | * | * | 7 |
| Kroenke, et al. | 2020 | - | * | * | * | * | * | * | * | 7 |
| Lara, et al. | 2020 | * | * | * | * | * | * | * | * | 8 |
| Lennartsson, et al. | 2021 | * | * | * | * | * | * | * | * | 8 |
| Liang, et al. | 2023 | * | - | * | * | ** | * | * | - | 7 |
| Lund, et al. | 2000 | * | * | * | * | ** | * | * | * | 9 |
| Luo, et al. | 2012 | * | * | * | * | ** | * | - | * | 8 |
| Maier, et al. | 1999 | * | * | - | * | - | * | * | * | 6 |
| Manemann, et al. | 2018 | - | * | * | * | * | * | - | * | 6 |
| Moreno – Tamayo, et al. | 2022 | * | * | - | * | * | * | * | * | 7 |
| Murberg, et al. | 2004 | - | * | - | * | * | * | * | * | 6 |
| Newall, et al. | 2013 | * | - | * | * | ** | - | * | * | 7 |
| Ng, et al. | 2015 | * | * | * | * | ** | * | * | * | 9 |
| Ng, et al. | 2021 | * | * | * | * | ** | * | * | - | 8 |
| Novak, et al. | 2020 | - | * | * | * | * | * | * | * | 7 |
| Novak, et al. | 2023 | * | * | * | * | ** | * | * | - | 8 |
| Olaya, et al. | 2017 | * | * | * | * | * | * | - | * | 7 |
| Olsen, et al. | 1991 | * | * | - | * | * | * | * | * | 7 |
| O'Súilleabháin, et al. | 2019 | - | * | * | * | - | * | * | * | 6 |
| Pennix, et al. | 1997 | - | * | * | * | * | * | - | * | 6 |
| Perissinotto, et al. | 2012 | * | * | * | * | * | * | * | * | 8 |
| Pimouguet, et al. | 2016 | * | * | * | * | * | * | - | * | 7 |
| Pitkala, et al. | 2004 | - | * | - | * | * | * | * | * | 6 |
| Renwick, et al. | 2020 | * | * | * | * | * | * | - | * | 7 |
| Rutledge, et al. | 2004 | - | * | * | * | * | * | * | * | 7 |
| Saito, et al. | 2012 | - | * | * | * | * | * | * | * | 7 |
| Saito, et al. | 2021 | * | * | * | * | * | * | * | * | 8 |
| Sakurai, et al. | 2019 | - | * | * | * | * | * | * | * | 7 |
| Scafato, et al. | 2008 | * | * | * | * | * | * | * | - | 7 |
| Schutter, et al. | 2021 | * | - | * | * | * | * | - | * | 6 |
| Seeman, et al. | 1993 | - | * | * | * | * | * | * | * | 7 |
| Shahtahmasebi, et al. | 1992 | * | * | - | * | * | * | * | - | 6 |
| Shiovitz-Ezra, et al. | 2010 | * | * | - | * | * | * | * | * | 7 |
| Smith, et al. | 2018 | * | * | * | * | * | * | * | * | 8 |
| Stek, et al. | 2005 | - | * | * | * | * | * | * | * | 7 |
| Steptoe, et al. | 2013 | * | * | * | * | * | * | * | * | 8 |
| Stressman, et al. | 2014 | * | * | - | * | * | * | * | * | 7 |
| Stringhini, et al. | 2018 | * | * | * | * | * | * | * | * | 8 |
| Tabue Teguo, et al. | 2016 | * | * | * | * | * | * | - | * | 7 |
| Takeuchi, et al. | 2018 | * | * | - | * | ** | - | * | * | 7 |
| Tilvis, et al. | 2011 | * | * | - | * | * | * | * | * | 7 |
| Tilvis, et al. (a) | 2012 | * | * | - | * | * | * | * | * | 7 |
| Tilvis, et al. (b) | 2012 | * | * | - | * | * | * | * | * | 7 |
| Wang, et al. | 2020 | * | * | * | * | * | * | * | - | 7 |
| Wang, et al. | 2022 | * | * | * | * | ** | * | * | * | 9 |
| Ward, et al. | 2021 | * | * | * | * | * | * | * | - | 7 |
| Wei, et al. | 2022 | * | * | - | * | ** | * | * | * | 8 |
| Yang, et al. | 2013 | * | * | * | * | * | * | * | * | 8 |
| Youm, et al. | 2021 | - | * | * | * | * | * | * | * | 7 |
| Yu, et al. | 2020 | - | * | * | * | ** | * | * | * | 8 |
| Yu, et al. | 2022 | * | * | - | * | ** | * | * | * | 8 |
| Yu, et al. | 2023 | * | * | * | * | ** | * | * | * | 9 |
| Zhen, et al. | 2015 | * | * | * | * | ** | - | * | * | 8 |

## Figure S1. Funnel plot of publications bias (p=0.0012) for the association between loneliness and all-cause mortality.

## Figure S2. Cumulative meta-analysis for the association between loneliness and all-cause mortality.

## Figure S3. Leave-one-out sensitivity meta-analysis for the association between loneliness and all-cause mortality.

## Figure S4. Forest plot for the association between loneliness and CVD and cancer mortality Notes: F=female, M=male

## Figure S5. Cumulative meta-analysis for the association between loneliness and CVD mortality.

## Figure S6. Leave-one-out sensitivity meta-analysis for the association between loneliness and CVD mortality.


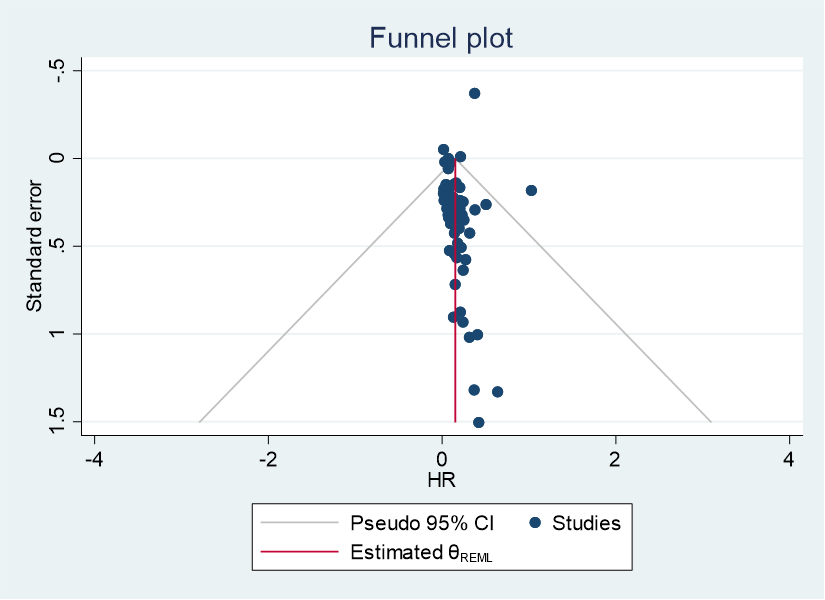


## Figure S7. Funnel plot of publications bias (p=0.000) for the association between social isolation and all-cause mortality.

## Figure S8. Cumulative meta-analysis for the association between social isolation and all-cause mortality.

Figure S9. Leave-one-out sensitivity meta-analysis for the association between social isolation and all-cause mortality.

## Figure S10. Forest plot for the association between social isolation and CVD, cancer, and other mortality Notes: F=female, M=male

Figure S11. Cumulative meta-analysis for the association between social isolation and CVD mortality. Notes: F=female, M=male

Figure S12. Leave-one-out sensitivity meta-analysis for the association between social isolation and CVD mortality. Notes: F=female, M=male

## Figure S13. Cumulative meta-analysis for the association between social isolation and cancer mortality. Notes: F=female, M=male

Figure S14. Leave-one-out sensitivity meta-analysis for the association between social isolation and cancer mortality. Notes: F=female, M=male

## Figure S15. Funnel plot of publications bias (p=0.4296) for the association between living alone and all-cause mortality.

## Figure S16. Cumulative meta-analysis for the association between living alone and all-cause mortality. Notes: F=female, M=male

## Figure S17. Leave-one-out sensitivity meta-analysis for the association between living alone and all-cause mortality. Notes: F=female, M=male

## Figure S18. Forest plot for the association between living alone and CVD, cancer, and other mortality

## Figure S19. Bubble plot for follow-up periods regarding the association between loneliness and all-cause mortality.

## Figure S20. Bubble plot for validated social network indexes, and NOS scale regarding the association between social isolation and all-cause mortality.


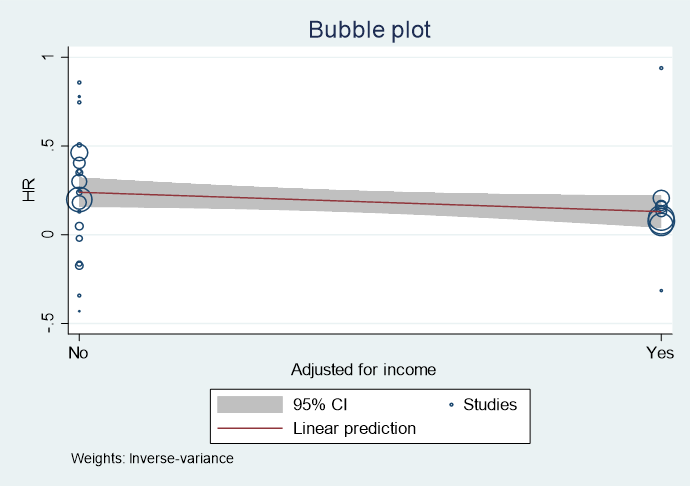


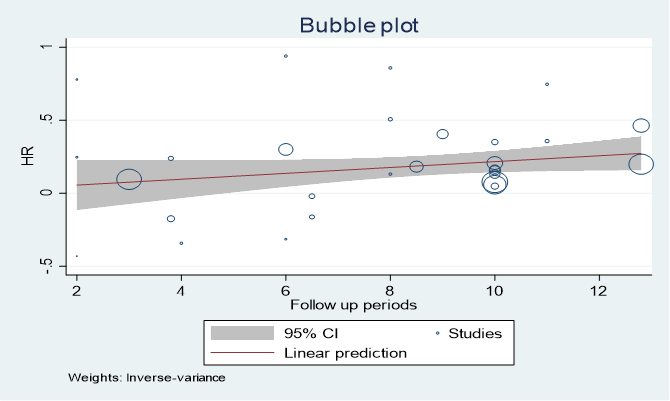


## Figure S21. Bubble plot for female (sex), adjusted for income, and follow-up periods regarding the association between living alone and all-cause mortality.
